# Mechanisms of ion selectivity and throughput in the mitochondrial calcium uniporter

**DOI:** 10.1126/sciadv.ade1516

**Published:** 2022-12-16

**Authors:** Bryce D. Delgado, Stephen B. Long

**Affiliations:** ^1^ Structural Biology Program, Memorial Sloan Kettering Cancer Center, 1275 York Avenue, New York, NY 10065, USA.; ^2^ Graduate Program in Biochemistry and Structural Biology, Cell and Developmental Biology, and Molecular Biology, Weill Cornell Medicine Graduate School of Medical Sciences, New York, NY 10065, USA.

## Abstract

The mitochondrial calcium uniporter, which regulates aerobic metabolism by catalyzing mitochondrial Ca^2+^ influx, is arguably the most selective ion channel known. The mechanisms for this exquisite Ca^2+^ selectivity have not been defined. Here, using a reconstituted system, we study the electrical properties of the channel’s minimal Ca^2+^-conducting complex, MCU-EMRE, from *Tribolium castaneum* to probe ion selectivity mechanisms. The wild-type *Tc*MCU-EMRE complex recapitulates hallmark electrophysiological properties of endogenous Uniporter channels. Through interrogation of pore-lining mutants, we find that a ring of glutamate residues, the “E-locus,” serves as the channel’s selectivity filter. Unexpectedly, a nearby “D-locus” at the mouth of the pore has diminutive influence on selectivity. Anomalous mole fraction effects indicate that multiple Ca^2+^ ions are accommodated within the E-locus. By facilitating ion-ion interactions, the E-locus engenders both exquisite Ca^2+^ selectivity and high ion throughput. Direct comparison with structural information yields the basis for selective Ca^2+^ conduction by the channel.

## Teaser

Electrical measurements of MCU-EMRE reveal mechanisms for selective Ca<sup>2+</sup> conduction by the mitochondrial Ca<sup>2+</sup> uniporter.

## INTRODUCTION

The mitochondrial calcium uniporter (the Uniporter) is a multisubunit Ca^2+^ channel complex that serves as the primary mechanism for Ca^2+^ uptake into mitochondria in higher eukaryotes (*1*, *2*). The Uniporter demonstrates exquisite selectivity for Ca^2+^, arguably making it the most selective ion channel known. The mechanisms for this selectivity and for its ability to conduct millions of Ca^2+^ ions per second are not well understood, in part, because mutations within the ion pore of the Uniporter that might affect these functions have yet to be studied using electrophysiology.

The Uniporter serves important biological functions. Mitochondrial calcium uptake through it regulates adenosine triphosphate (ATP) production to meet energy demands, shapes cytosolic calcium signals, and controls a mitochondrial permeability transition that, when activated, leads to mitochondrial stress and cell death (*2*). Mice lacking the pore-forming subunit of the Uniporter have diminished ability to respond to perform strenuous work (*3*). Recently, it has been shown that the Uniporter coordinates ATP production with action potential firing in neurons in real time (*4*). The Uniporter is also implicated in regulating cell-intrinsic antimicrobial response (*5*), cardiac hypertrophy and hereditary cardiomyopathy (*6*), memory formation (*7*), and muscle myopathies (*8*). A negative electrical potential (~−160 mV) of the mitochondrial matrix relative to the cytosol, which is established by the H^+^ gradient, serves as a driving force for the uptake of cations from the cytosol. The selectivity of the Uniporter for Ca^2+^ is of paramount importance—K^+^ and other cations outnumber Ca^2+^ by approximately 1 million–fold. Without high selectivity for Ca^2+^, substantial influx of K^+^ would reduce the proton motive force used for ATP synthesis.

The transmembrane proteins MCU and EMRE are the core ion channel–forming components of the Uniporter (*9*, *10*). It has been shown, for example, that coexpression of MCU and EMRE in yeast, which do not contain an endogenous Uniporter, is sufficient to catalyze mitochondrial Ca^2+^ uptake (*11*). The other known components of Uniporters in higher eukaryotes, which generally serve regulatory roles, are the proteins MICU1 to MICU3 (*12*–*14*), MCUR1 (*15*–*17*), and MCU_b_ (*18*). The MICU1 to MICU3 proteins, for example, confer Ca^2+^-dependent activation to the channel whereby these subunits reduce Ca^2+^ flux at low resting concentrations of Ca^2+^ (below 1 μM) by occluding the ion conduction pathway and set a threshold for Ca^2+^-dependent activation (*13*, *19*–*23*).

**Fig. 1.**
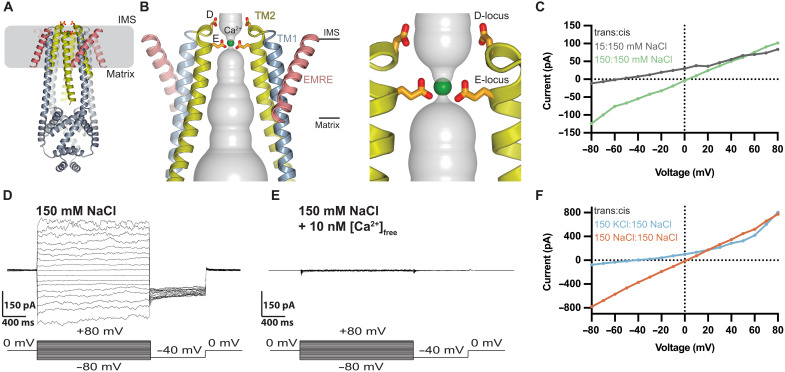
Monovalent currents of *Tc*MCU-EMRE. (**A** and **B**) Three-dimensional cryo-EM structure of *Tc*MCU-EMRE (Protein Data Bank: 6X4S, cartoon representation). The TM2 helices are yellow, EMRE is pink, and the remainder of the channel is gray. Ca^2+^ is depicted as a green sphere. (B) View and close-up (right) of the transmembrane region and acidic loci. Two subunits are shown (the other two are removed for clarity). The D-locus and E-locus residues are drawn as sticks. The gray surface represents the ion pore. Approximate boundaries of the membrane are indicated. (**C** to **F**) Bilayer experiments using purified *Tc*MCU-EMRE. (C) *Tc*MCU-EMRE is cation selective. *I*-*V* relationships are plotted for symmetric (150 mM NaCl:150 mM NaCl; green) and asymmetric (15 mM NaCl:150 mM NaCl; gray) salt concentrations under divalent-free conditions. Buffer components are listed as trans:cis. A reversal potential of −57.4 ± 2.7 mV for the asymmetric condition agrees with the Nernst potential (−58 mV) for a perfectly cation-selective ion channel. (D) Macroscopic Na^+^ currents under divalent-free conditions (symmetric 150 mM NaCl). The voltage protocol is shown at the bottom. To obtain *I*-*V* relationships similar to those in (C), mean currents of the test pulses were calculated over 1.7 s. (E) Nanomolar concentrations of Ca^2+^ inhibit Na^+^ currents. [Ca^2+^]_free_ (10 nM) was added to the same bilayer from (D), and the voltage protocol was repeated. (F) *Tc*MCU-EMRE displays selectivity for Na^+^ over K^+^. Asymmetric conditions (150 mM KCl:150 mM NaCl; blue) exhibit a reversal potential of −42 ± 5 mV, indicating a *P*_K_/*P*_Na_ permeability ratio of 0.22 ± 0.06.

To date, 10 x-ray or cryo–electron microscopy (cryo-EM) structures of metazoan MCU-EMRE complexes or of fungal MCU complexes have been determined (*23*–*28*). The structures reveal a conserved architecture of the channel, but no structural similarity to other proteins, which limits insight into the Ca^2+^ selectivity mechanisms that can be drawn from other channels. The structures reveal that a tetrameric assembly of MCU subunits forms an ion pore along the axis of symmetry between the subunits. The EMRE subunits, which may be only present in metazoan organisms (*9*), are positioned at the periphery of the transmembrane region and do not appear to contribute to the walls lining the pore in the structures (*28*, *29*). Subtle conformational differences among some of the MCU-EMRE structures in the region closest to the mitochondrial matrix could represent different gating conformations of the channel (*28*, *29*). Of note for the current work, the conformation of the pore closest to the mitochondrial intermembrane space (IMS) is indistinguishable among all these structures. This region includes a conserved sequence motif containing two acidic amino acids (D261 and E264, human MCU numbering) that has been postulated to function as the selectivity filter responsible for the channel’s remarkable Ca^2+^ selectivity (*24*–*27*, *30*). The structures show that this region is α-helical, which was initially unexpected, as a portion was proposed to be in a loop (*18*, *30*, *31*). In the structures, D261 and E264 from each of the four subunits face the pore, creating two acidic rings at the mouth of the pore (Fig. 1, A and B, and fig. S1). These amino acids, which we refer to as the D-locus and E-locus, are perfectly conserved among all MCU proteins identified to date. Mutation of these residues has been shown to alter or eliminate the ability of the channel to catalyze Ca^2+^ uptake into mitochondria (*30*), suggesting that they have important roles in channel function. However, the influences of these residues on ion selectivity have not been evaluated by electrophysiological studies of mutants.

Electrophysiological studies of the endogenous Uniporter from mammalian cells, made from mitochondria stripped of their outer membrane (so-called “mitoplasts”), show that, similar to some other cellular Ca^2+^ channels, the Uniporter conducts monovalent cations, such as Na^+^, when Ca^2+^ is removed (*1*). Ca^2+^ potently inhibits these Na^+^ currents with a dissociation constant (*K*_d_) of <2 nM, indicating that the Uniporter contains a high-affinity binding site for Ca^2+^ (*1*). However, the Uniporter does not appreciably conduct Ca^2+^ until much higher (~mM) levels of Ca^2+^ are present, and Ca^2+^ currents do not saturate until approximately 80 mM (*1*). Experiments showing current measurements through individual channels reveal a single-channel conductivity for Ca^2+^ of 2.6 to 5.2 pS at −160 mV (*1*), indicating that the Uniporter is highly conductive and can permit approximately 10^6^ Ca^2+^ ions to flow through an individual channel per second. The high-affinity binding of Ca^2+^ and the high throughput of Ca^2+^ are paradoxical—a binding site with such high affinity would typically have an off-rate that is several orders of magnitude slower than the rate at which the channel has been shown to catalyze Ca^2+^ conduction. In this study, we sought to address this seeming paradox by investigating the roles of the D- and E-loci using electrophysiological studies of channel mutants.

While mitoplast recordings have yielded insight into the properties of endogenous Uniporter channels (*1*, *9*, *16*), they are very technically challenging (*32*). Mitoplast recordings are also complicated by the heterogeneity of the protein components present, potential mitochondrial toxicities when mutations are introduced, and the inability to change the solution on the matrix side of the membrane during the experiment, among other considerations. For these reasons, we sought to use an alternative approach to study the electrical properties of mutations made to the D- and E-loci. Two-electrode voltage clamp experiments of channel subunits expressed in frog oocytes present one alternative. Such experiments have been used to demonstrate that expression of an MCU-EMRE fusion protein in the oocyte membrane generates a channel with electrical properties similar to the mitochondrial uniporter (*33*). While more technically tractable than mitoplast recordings, two-electrode voltage clamp experiments are complicated by the inability to change the solution inside the oocyte during the experiment and by the presence of endogenous Ca^2+^-activated chloride channels in their membranes, which could, for example, cloud the effects of introduced mutations that alter ion selectivity. Nevertheless, it has been shown using two-electrode voltage clamp experiments that mutation of the E-locus to alanine in an MCU-EMRE fusion construct eliminates the ability of the channel to conduct Ca^2+^ (*33*), which is in agreement with previous mitochondrial Ca^2+^ uptake measurements (*24*, *30*) and suggests that the E-locus has an important role in channel function.

We chose a reductionist approach to interrogate the ion selectivity properties of purified MCU-EMRE complexes and their mutants in a reconstituted lipid bilayer system. This system allows complete chemical control of the lipid and protein constituents, as well as the ability to change the ionic compositions of the solutions bathing each side of the membrane during the experiment (*34*). With this system, the ion selectivity properties of mutants can be observed directly without concerns that the effects might stem from endogenous ion transport proteins in mitochondria or oocytes. Previous attempts to study MCU/EMRE using bilayer electrophysiology (*18*, *31*, *35*) were, however, met with skepticism (*10*, *33*). For example, previous electrical recordings were made at the single-channel level and inconsistent with single-channel recordings of endogenous channels. The ability to interrogate multiple channels simultaneously, so-called “macroscopic” electrical recordings, when using a reconstituted system is important to establish that the recordings emanate from the protein of interest, in part, because single-channel electrical signals may arise from trace contaminants, even when using a highly purified protein (*36*). In addition, most previous attempts to electrically characterize purified channel components were performed before the discovery that EMRE is a necessary component for Ca^2+^ permeation through MCU, which casts doubt that MCU was responsible for the observed currents (*18*, *31*). In this study, we first sought to evaluate whether a purified MCU-EMRE complex could recapitulate macroscopic electrical hallmarks of endogenous Uniporter channels. Upon demonstrating this, we then investigated the effects of mutations of the D- and E-loci to interrogate the mechanisms for the channel’s superlative selectivity for Ca^2+^ and high throughput.

To select the MCU-EMRE protein construct for the functional studies presented here, we leaned on previous work, in which our laboratory determined cryo-EM structures of the MCU-EMRE complex from the red flour beetle *Tribolium castaneum*, which we refer to as *Tc*MCU-EMRE (*22*, *29*). This complex exhibited superior biochemical stability in comparison to human and other metazoan MCU-EMRE complexes that we evaluated. The *Tc*MCU and *Tc*EMRE subunits share 77 and 48% identity with the human counterparts within their transmembrane domains. Similar to the human Uniporter, EMRE is required for Ca^2+^ uptake by *Tc*MCU (*29*). Stemming from the observation that functional channels in mitochondria can be formed by covalent fusion of MCU and EMRE subunits (*37*), the MCU and EMRE proteins were connected with a flexible peptide linker for structural and functional studies (*29*). The N-terminal domain (NTD) of MCU (amino acids 1 to 168) was also removed; we and others have found that the NTD is not required for Ca^2+^ uptake into mitochondria (*24*, *28*, *29*, *38*, *39*) and that higher-resolution cryo-EM structures can be determined without the NTD owing to its flexible connection with the pore domain (*22*, *29*). We have previously shown that this *Tc*MCU-EMRE construct catalyzes Ca^2+^ uptake into mitochondria when heterologously expressed in mammalian cells and that purified *Tc*MCU-EMRE protein catalyzes Ca^2+^ uptake into liposomes (*29*). We suspect that the superior biochemical stability of the *Tc*MCU-EMRE construct facilitates functional studies of the purified protein. These attributes inspired us to pursue the electrical recordings of purified *Tc*MCU-EMRE presented here, which also allow for direct comparison with its three-dimensional structure.

Using the reconstituted bilayer system, we determined that purified *Tc*MCU-EMRE protein recapitulates hallmark electrical properties of endogenous Uniporters. By making mutations of the E- and D-loci and by studying the electrical properties of the modified channels, we identify that the E-locus serves as the channel’s selectivity filter. The D-locus has only minor influence on ion selectivity. Using noise analysis, we characterize single-channel properties of the wild-type and mutant channels and identify that the E-locus enables rapid ion conduction. From anomalous mole fraction effects, we observe evidence for ion-ion interactions within the E-locus. We conclude that the E-locus confers exquisite Ca^2+^ selectivity and catalyzes rapid ion conduction by accommodating multiple interacting Ca^2+^ ions within this single locus.

## RESULTS

### A reconstituted MCU-EMRE complex recapitulates electrical properties of the Uniporter

The *Tc*MCU-EMRE protein was purified from mammalian cells as previously described (*29*) and then reconstituted into liposomes (see fig. S2 and Materials and Methods). Cardiolipin was included in the liposomes following the results of our previous flux assay experiments that indicated its importance for channel function (*29*). The reconstituted protein was studied using planar lipid bilayer electrophysiology, which allowed for the precise control of ionic, lipid, and protein composition (*34*). Using macroscopic electrical recordings (from numerous channels simultaneously), we first sought to characterize whether the purified sample exhibited electrical properties previously observed for endogenous Uniporters. These properties include Na^+^ permeability under divalent-free conditions, potent block of Na^+^ currents by Ca^2+^, robust Ca^2+^ permeation at higher levels of Ca^2+^, inhibition by ruthenium red (RuRed), current rectification, and selectivity for Ca^2+^ following the conductivity sequence: Ca^2+^ ~ Sr^2+^ >> Mn^2+^ ~ Ba^2+^ > Mg^2+^ (*1*).

We observed robust Na^+^ currents under divalent-free conditions (Fig. 1, C and D). *Tc*MCU-EMRE displayed essentially perfect selectivity for Na^+^ over Cl^−^ as evidenced by a reversal potential at the Nernst potential when an asymmetric concentration of NaCl was used across the membrane (Fig. 1C). Unlike most Ca^2+^ channels, the Uniporter displays a preference for Na^+^ over K^+^ under divalent-free conditions (*1*, *40*). This property is thought to relate to the slightly smaller size of Na^+^ and to be an indication of narrow dimensions in the Uniporter’s pore (*1*). *Tc*MCU-EMRE exhibited this preference with a permeability ratio (*P*_K_/*P*_Na_) of 0.22 ± 0.06 (Fig. 1F). We found that monovalent cation permeation properties of the Uniporter are recapitulated by purified *Tc*MCU-EMRE.

While Na^+^ currents through many Ca^2+^ channels are blocked by micromolar levels of Ca^2+^, a notable property of the Uniporter is the ability for low nanomolar levels of Ca^2+^ to block such currents (*1*, *40*, *41*). The high-affinity interaction of Ca^2+^ in the pore is thought to relate to the Uniporter’s superlative selectivity for Ca^2+^ (*1*). Similar to endogenous Uniporters (*1*), we found that Na^+^ currents through *Tc*MCU-EMRE were inhibited by nanomolar levels of Ca^2+^ (Fig. 1E).

**Fig. 2.**
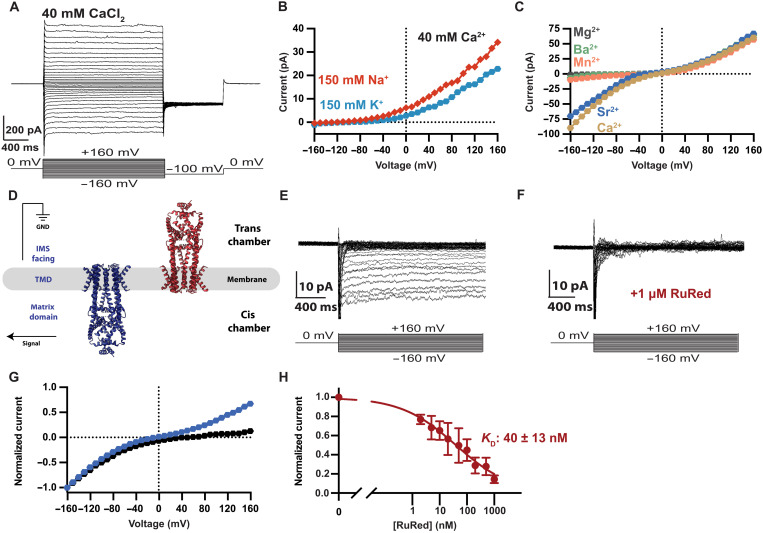
Ca^2+^ selectivity and RuRed inhibition of *Tc*MCU-EMRE. (**A**) Macroscopic Ca^2+^ currents of *Tc*MCU-EMRE in a planar lipid bilayer. Symmetric solutions containing 40 mM CaCl_2_ were used. The voltage protocol is shown at the bottom. (**B**) Selectivity for Ca^2+^ over Na^+^ and K^+^. Representative *I*-*V* curves are shown for bi-ionic conditions (150 mM KCl or NaCl in the trans chamber:40 mM CaCl_2_ and 150 mM NaCl in the cis chamber). (**C**) Divalent cation selectivity. Representative *I*-*V* curves for symmetric 40 mM CaCl_2_ (light brown) and bi-ionic (40 mM XCl_2_ trans:40 mM CaCl_2_ cis) solutions. (**D**) Schematic of the planar lipid bilayer system, showing channels oriented in both directions. TMD, transmembrane domain; GND, electrical ground. (**E**) *Tc*MCU-EMRE exhibits rectification for Ca^2+^ currents. Representative Ca^2+^ current traces using symmetric 40 mM CaCl_2_ with 1 μM RuRed in the cis chamber. (**F**) Using the same membrane as in (E), the addition of 1 μM RuRed to the trans chamber inhibits Ca^2+^ currents. (**G**) *I*-*V* relationships showing rectification of Ca^2+^ currents (symmetric 40 mM CaCl_2_). Blue trace is without RuRed; black trace is with 1 μM RuRed in the cis chamber [from (E)]. For comparison, the *I*-*V* relationships were normalized by dividing by the mean current at −160 mV. Before normalization, each experiment elicited ~30 pA of current at −160 mV. (**H**) Binding affinity of RuRed. Normalized mean Ca^2+^ currents observed at −160 mV (symmetric 40 mM CaCl_2_) in the presence of different concentrations of RuRed in the trans chamber were plotted as shown. Currents were normalized by dividing by the uninhibited value and fit as described in Materials and Methods. The Hill coefficient of the fit was 0.4 ± 0.1. Error bars represent the SEM from three independent experiments. 1 μM RuRed was used in the cis chamber to block channels with their IMS side facing the cis chamber.

Although nanomolar levels of Ca^2+^ block monovalent cation permeation in the Uniporter, the endogenous channel does not efficiently conduct Ca^2+^ until millimolar levels of Ca^2+^ are present (*1*). These two apparent affinities for Ca^2+^ in the Uniporter, nanomolar block and millimolar conduction, hint that ion conduction could involve multiple Ca^2+^ ions within the pore. In voltage-dependent calcium (Ca_V_) or voltage-dependent potassium (K_V_) channels, multiple interacting ions have been shown to overcome the seeming paradox of tight ion binding and high flux (*42*, *43*). *Tc*MCU-EMRE exhibited an analogous pattern—nanomolar block of Na^+^ currents by Ca^2+^ and robust Ca^2+^ current through the channel when 40 mM Ca^2+^ was present (Figs. 1, D and E, and 2A). Bi-ionic conditions with 40 mM Ca^2+^ on one side and 150 mM Na^+^ or K^+^ on the other only displayed currents for Ca^2+^, indicating near-perfect selectivity for Ca^2+^ over monovalent cations (Fig. 2B).

The macroscopic Ca^2+^ currents were approximately 10 times smaller than the macroscopic Na^+^ currents recorded under divalent-free conditions from the same membrane (at near-saturating levels of cations; 40 mM Ca^2+^ or 150 mM Na^+^; fig. S3). Lower macroscopic Ca^2+^ currents in comparison to Na^+^ could be due to reduced flow of ions through each open channel (lower single-channel conduction), reduced probability of the channels adopting an open state (lower open probability), or a combination of these. We observed a substantial amount of current fluctuation (also referred to as “noise”) in the macroscopic current traces for Na^+^ (Fig. 1D). The fluctuations are presumably due to the collection of channels in the membrane opening and closing stochastically. The fluctuations were notably reduced for Ca^2+^ currents from the same membrane (e.g., one that contains the same number of ion channels), which is another indication of a change in the open-probability and/or single-channel conductance of the channel when Ca^2+^ is the charge carrier in comparison to Na^+^ (fig. S3). We used analyses of such fluctuations to estimate the single-channel properties of *Tc*MCU-EMRE, as described later.

Endogenous Uniporter channels have a divalent conductivity sequence that distinguishes them from most other Ca^2+^ channels (*1*). For the Uniporter, Ca^2+^ and Sr^2+^ are similarly conductive, but Mn^2+^, Ba^2+^, and Mg^2+^ are hardly conductive (Ca^2+^ ~ Sr^2+^ >> Mn^2+^ ~ Ba^2+^ > Mg^2+^). The strong preference for Ca^2+^ over Ba^2+^ is unusual among Ca^2+^ channels. For example, Ca_V_ channels, the calcium release–activated calcium channel, the inositol triphosphate receptor, and the ryanodine receptor exhibit higher conductance for Ba^2+^ compared to Ca^2+^ (*44*–*48*). While the transient receptor potential channel TRPV6 shows selectivity for Ca^2+^ over Ba^2+^, similar to the Uniporter, it displays low conductivity to Sr^2+^ (*49*, *50*), in contrast to the Uniporter, for which Sr^2+^ and Ca^2+^ are similarly conductive. Thus, the distinct conductivity sequence is a hallmark of the Uniporter.

Using bi-ionic conditions, with 40 mM Ca^2+^ on one side of the membrane and 40 mM of a test divalent cation on the other, we observe that *Tc*MCU-EMRE exhibits the same Ca^2+^ ~ Sr^2+^ >> Mn^2+^ ~ Ba^2+^ > Mg^2+^ conductivity sequence as endogenous Uniporter channels (Fig. 2C). This agreement inspires further confidence that *Tc*MCU-EMRE recapitulates fundamental properties of the Uniporter. Previously, low conductance of Mn^2+^ had been attributed, in part, to an inability of Mn^2+^ to bind to the EF-hands of MICU proteins that release from their inhibitory conformation at the mouth of the ion pore (*51*, *52*). Our results indicate that divalent selectivity is an inherent property of the pore of the channel, conferred by MCU and EMRE subunits, and independent of MICU proteins or other protein components of the Uniporter that are not present in our reconstituted system.

In the reconstituted system, purified *Tc*MCU-EMRE channels could be positioned in the bilayer in two possible orientations (*34*), with the IMS side of their pore facing either the cis or the trans compartment of the apparatus (Fig. 2D). Although endogenous Uniporter channels are strong rectifiers, which preferentially catalyze the flow of Ca^2+^ into the mitochondrial matrix (*1*), we observe robust Ca^2+^ current at both positive and negative voltages (Fig. 2A). The current-voltage (*I*-*V*) curve is sigmoidal (Fig. 2, C and G), however, which would occur if rectifier channels were present in both orientations. We reasoned that if we could preferentially inhibit channels facing in one direction, we might observe current rectification from channels facing in the other direction. Ca^2+^ currents through endogenous Uniporters are blocked by the cationic molecule RuRed when it is added on the IMS side of the membrane (*1*). Its binding site has been attributed to the D-locus on the IMS entrance of the ion pore (*53*), which suggests that it is a sided blocker. We found that the application of 1 μM RuRed to the cis side of the bilayer, which would inhibit channels with their IMS side facing the cis chamber, nearly eliminated Ca^2+^ currents at positive voltages (Fig. 2, E and G). Inhibition of these channels divulged the strong inward Ca^2+^ current rectification by *Tc*MCU-EMRE channels oriented in the other direction, e.g., with their IMS side facing the trans chamber (Fig. 2, D, E, and G). By titrating the concentration of RuRed, we found that it inhibits *Tc*MCU-EMRE with a median inhibitory concentration (IC_50_) of approximately 40 nM (Fig. 2, F and H), which is in accord with the potency observed for endogenous Uniporters (*1*). Our results indicate that rectification is an inherent property of the MCU-EMRE complex and that rectification does not require additional subunits.

The RuRed experiments introduced the technical complication that we found it difficult to wash the compound from the apparatus following the experiment; consequently, we did not use RuRed in other experiments. The presence of active channels facing in both orientations does not alter the conclusions drawn from the experiments presented in this work. The strong rectification observed for Ca^2+^ provides evidence that currents observed at negative voltages originate from channels with their matrix side facing the cis chamber and those observed at positive voltages originate from channels facing in the other orientation.

In summary, we found that purified *Tc*MCU-EMRE protein recapitulated hallmark electrical properties of the Uniporter in our experimental bilayer system. These include Na^+^ permeability under divalent-free conditions, preference for Na^+^ over K^+^, nanomolar-affinity blockade of Na^+^ currents by Ca^2+^, robust Ca^2+^ permeation at millimolar levels, inhibition by RuRed, current rectification, and a Ca^2+^ ~ Sr^2+^ >> Mn^2+^ ~ Ba^2+^ > Mg^2+^ conductivity sequence.

### Mutagenesis identifies the E-locus as the high-affinity site

**Fig. 3.**
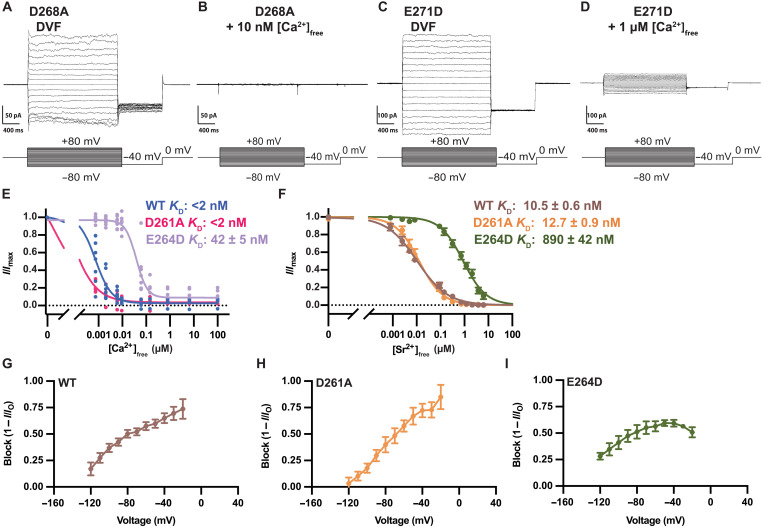
E-locus is the high-affinity binding site for Ca^2+^. (**A** and **B**) Similar to wild-type (WT) *Tc*MCU-EMRE, monovalent cation currents through the D→A mutant are inhibited by nanomolar levels of Ca^2+^. Representative current traces of the D→A (D261A) mutant using symmetric 150 mM NaCl under divalent-free conditions, denoted “DVF” (A), and in the presence of symmetric 10 nM [Ca^2+^]_free_ (B). The same conditions and voltage protocol were used as for the wild-type *Tc*MCU-EMRE shown in Fig. 1. (**C** and **D**) Representative current traces of the E→D (E264D) mutant using symmetric 150 mM NaCl under divalent-free conditions (C) and in the presence of symmetric 1 μM [Ca^2+^]_free_ (D). (**E**) Dose-response curves for inhibition of Na^+^ currents by Ca^2+^ for the wild-type and mutant channels from experiments analogous to those in (A) to (D) using a range of [Ca^2+^]_free_. Currents (at −80 mV) were normalized by dividing by the uninhibited (divalent-free) value (*I*_max_). Each point represents an experiment using the indicated concentration of [Ca^2+^]_free_. Upper limits for *K*_d_ are given for the wild-type and D→A mutant channels because the affinity is greater than the buffering capacity of EGTA. (**F**) Dose-response curves for inhibition of Na^+^ currents by Sr^2+^. Experiments were analogous to those described above, using Sr^2+^ instead of Ca^2+^. (**G** to **I**) Voltage dependence of Sr^2+^ blockade. The extent of block of Na^+^ currents at a given concentration of Sr^2+^ is a function of the applied voltage. Curves for wild-type and D→A channels are shown using 10 nM [Sr^2+^]_free_. The curve for the E→D mutant is using 1 μM [Sr^2+^]_free_. Block is calculated by dividing the current at the test voltage in the presence of Sr^2+^ (*I*) by the current at the test voltage in the absence of divalent ions (*I*_O_) and subtracting this fraction from 1.

After determining that purified *Tc*MCU-EMRE protein exhibited electrical properties of the Uniporter, we turned to study the effect of mutations of the acidic loci in the putative selectivity filter region. Among several amino acid substitutions tried, many resulted in low protein expression or poor biochemical stability (Materials and Methods). Mutation of the D-locus to alanine (D261A) or the E-locus to aspartic acid (E264D) (Fig. 1B), which will subsequently be referred to as the D→A and E→D mutants, respectively, yielded proteins that could be purified (fig. S2). The size exclusion chromatography profiles of the purified mutants were similar to that of the wild-type protein, suggesting that the mutations did not disrupt the overall fold of the channel. The interrogation of purified D→A and E→D mutants in the bilayer indicated that both yielded Na^+^ currents under divalent-free conditions that could be blocked by Ca^2+^ (Fig. 3, A to D). Similar to the wild-type channel, both mutants displayed strong selectivity for cations over anions as evaluated by reversal potentials near the Nernst potential for Na^+^ selectivity using asymmetric concentrations of NaCl (fig. S4). These properties are further indications that the structure of the mutants generally corresponds to the wild-type channel.

By titrating the amount of Ca^2+^, we found that Ca^2+^ blocked the Na^+^ currents observed for wild-type *Tc*MCU-EMRE at −80 mV with a *K*_d_ value of <2 nM (Fig. 3E). The D→A mutant displayed an analogously high-affinity block of Na^+^ currents (*K*_d_ < 2 nM) (Fig. 3E). The E→D mutant, on the other hand, displayed incomplete block and markedly weaker affinity for Ca^2+^ (*K*_d_ ~ 42 nM) (Fig. 3, C to E).

To further investigate the E- and D-loci, we quantified the ability of Sr^2+^ to block Na^+^ currents (Fig. 3F). Sr^2+^ was chosen because previous mitoplast recordings of the Uniporter (*1*) and our studies show that it has similar conductivity properties to Ca^2+^, which suggests that it interacts with the pore in analogous ways. We found that Na^+^ currents through wild-type *Tc*MCU-EMRE were blocked by Sr^2+^ with a *K*_d_ of 10.5 ± 0.6 nM. The somewhat weaker affinity for Sr^2+^ in comparison to Ca^2+^ provided the technical advantage that the divalent cation concentration could be more precisely manipulated by a buffering system, which enabled us to define the effects of the mutagenesis more fully. Na^+^ currents through both mutants were blocked by Sr^2+^, with the D→A mutant exhibiting a *K*_d_ value of 12.7 ± 0.9 nM, whereas the E→D mutant displayed a markedly weaker *K*_d_ of 890 ± 42 nM (Fig. 3F). Thus, for both Ca^2+^ and Sr^2+^, a high-affinity block of Na^+^ currents was observed in the D→A mutant, but the more conservative E→D mutant yielded dramatically weaker affinity. These results suggest that the E-locus represents the high-affinity binding site for Ca^2+^/Sr^2+^ and indicate that the D-locus is not a component of it.

We found that the potency of block of the wild-type channel by a concentration of Sr^2+^ near its *K*_d_ was voltage dependent. Stronger (more negative) voltages resulted in a lower extent of block, whereas voltages closer to neutral yielded more block (Fig. 3G). The observed voltage dependence indicates that the blocking site is located within the electric field. The diminished block at stronger voltages suggests that a blocking cation can be pulled from the blocking site into the pore by the electric field, in a so-called “punch-through” effect (*54*–*57*). This is evidence that a Sr^2+^ (and presumably a Ca^2+^) ion bound in the blocking site experiences a force due to an electric field across the membrane.

The D→A mutant displayed the same pattern, with a voltage dependence that was analogous to that of the wild-type channel (Fig. 3H). The similarities between the wild type and D→A mutant regarding block by Ca^2+^ and Sr^2+^ suggest that the blocking site within the E-locus is unperturbed by the D→A mutation at the mouth of the pore. The E→D mutant, on the other hand, displayed relatively modest voltage dependence of block, which provides further evidence that the high-affinity blocking site present in the wild-type and D→A mutant channels is the E-locus (Fig. 3I).

### The E-locus is the selectivity filter

**Fig. 4.**
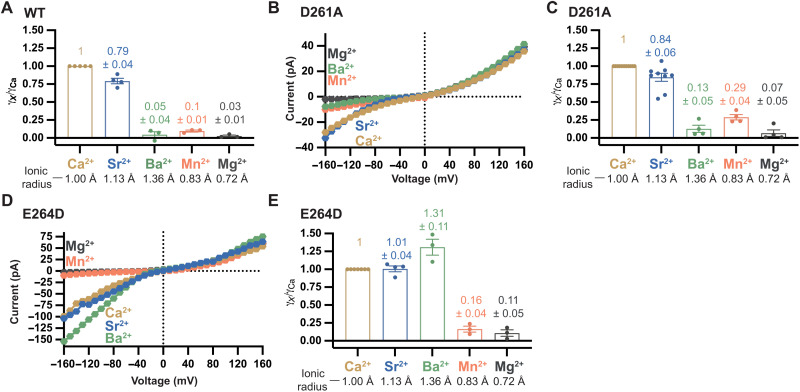
Divalent selectivity of mutant channels. (**A**) Relative conductance ratios for wild-type *Tc*MCU-EMRE deduced from *I*-*V* relationships as those shown in Fig. 2C. Conductance ratios were determined by dividing the mean current observed for a test cation at −160 mV by the value observed for Ca^2+^ from the same membrane. Points within each bar represent different experiments (and different membranes). Effective ionic radii for ions are indicated below the graph (*101*). (**B** and **C**) Divalent selectivity properties of the D→A mutant. (B) Representative *I*-*V* curves for symmetric 40 mM CaCl_2_ (light brown) and bi-ionic (40 mM XCl_2_ trans and 40 mM CaCl_2_ cis) solutions. (C) Conductance ratios among divalent cations are shown, calculated as in (A). (**D** and **E**) Divalent selectivity properties of the E→D mutant, showing representative *I*-*V* curves (D) and conductance ratios (E), determined as indicated above.

In addition to conducting Na^+^ under divalent-free conditions, both the D→A and E→D mutant channels conducted Ca^2+^ when millimolar levels were present (Fig. 4, B and D, and fig. S5). We evaluated the divalent cation selectivity properties using bi-ionic conditions, with Ca^2+^ on one side of the membrane and a test divalent cation on the other. Notably, the D→A mutant exhibits the same divalent conductivity sequence as the wild-type channel, Ca^2+^ ~ Sr^2+^ >> Mn^2+^ ~ Ba^2+^ > Mg^2+^ (Fig. 4B). Current recordings suggested that this mutant may have slightly higher relative conductivities of Mn^2+^ and Ba^2+^ than the wild-type channel (Figs. 2C and 4, A to C). For the D→A mutant and the wild-type channel, the conductivity sequence correlates with ionic radii. The highly conductive Ca^2+^ and Sr^2+^ ions have similar ionic radii (Fig. 4, A and C), 1.00 and 1.13 Å, respectively. By contrast,Ba^2+^, with a larger ionic radius (1.36 Å), or Mn^2+^ and Mg^2+^, with smaller ionic radii (0.83 and 0.72 Å, respectively), are much less conductive. The E→D mutant displayed a different divalent conductivity sequence from the wild-type pore, in which Ba^2+^ is more conductive than Ca^2+^ (Ba^2+^ > Ca^2+^ ~ Sr^2+^ >> Mn^2+^ ~ Mg^2+^) (Fig. 4, D and E). The increased conductivity of the larger Ba^2+^ ion suggests that the selectivity filter is made wider by the mutation. These results show that the ion selectivity of the relatively conservative E→D mutant of the E-locus is radically different from that of the wild-type channel. On the other hand, the chemically pronounced D→A mutation of the D-locus yields a channel with divalent cation selectivity properties highly similar to that of the wild-type channel.

**Fig. 5.**
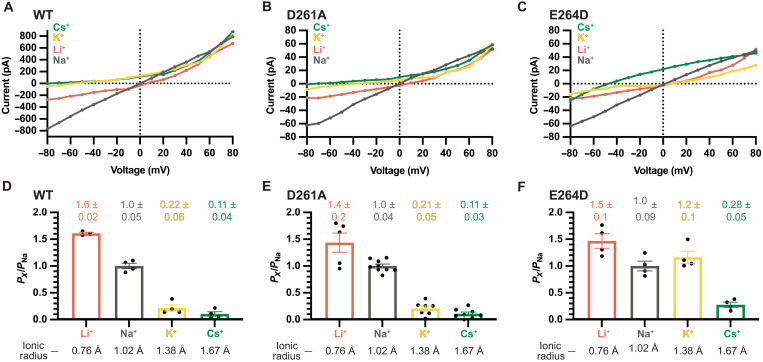
Monovalent selectivity of *Tc*MCU-EMRE and mutants. (**A** to **C**) Representative *I*-*V* relationships of *Tc*MCU-EMRE and mutants under divalent-free conditions. The trans chamber contained 150 mM XCl test salt, as indicated, and the cis chamber contained 150 mM NaCl. (**D** to **F**) Relative permeability ratios (*P_X_*/*P*_Na_) among monovalent cations for *Tc*MCU-EMRE and mutants. As described in Materials and Methods, permeability ratios were determined from reversal potentials. Points within the bars represent different experiments from different membranes. Error bars represent the SEM. Effective ionic radii for the cations are indicated (*101*).

We next sought to study the monovalent cation permeability properties of the wild-type and mutant channels under divalent-free conditions to further characterize the physiochemical properties of the ion pore. Using bi-ionic conditions, with Na^+^ on one side and a monovalent test cation on the other, *Tc*MCU-EMRE exhibited a permeability sequence of Li^+^ > Na^+^ > K^+^ > Cs^+^ as determined by reversal potentials (Fig. 5, A and D, and fig. S6). This monovalent permeability sequence reflects the Eisenman type XI highest field strength selectivity sequence, indicating that the selectivity filter of the open pore interacts strongly with ions at a distance of less than 1.5 Å (*58*). In such a pore, the ion directly interacts with the surrounding pore, indicating that it becomes at least partially dehydrated during conduction. The permeability of an ion can be thought of as the ability of that ion to enter the pore, whereas conductivity relates to the rate at which an ion passes through the pore (*55*). The permeability sequence correlates with ionic radii: Smaller monovalent cations (e.g., Li^+^ and Na^+^) are more permeable than larger ones; that is, smaller ones more easily enter the pore (Fig. 5D). The correlation with size suggests that ions the size of K^+^ or larger are too large to fit optimally. *I*-*V* relationships show that the conductivity sequence of *Tc*MCU-EMRE is slightly different from the permeability sequence, with Na^+^ exhibiting higher conductivity than Li^+^: Na^+^ > Li^+^ > K^+^ > Cs^+^ (Fig. 5A). Na^+^, with an ionic radius of 1.02 Å, seems optimally sized to pass through the filter. Together, the monovalent cation selectivity results suggest that the selectivity filter acts in a size-selective manner. Na^+^ with an ionic radius of 1.02 Å conducts most easily, whereas the conductivity of the smaller Li^+^ ion (ionic radius of 0.76 Å) is less, and larger K^+^ and Cs^+^ ions (ionic radii of 1.38 and 1.67 Å, respectively) are restricted. This size-based selection is consistent with the divalent cation selectivity results, in which Ca^2+^ and Sr^2+^, with ionic radii of 1.00 and 1.13 Å, respectively, are highly conductive, whereas larger or smaller ions are much less so (Fig. 4A). A cation with an ionic radius of approximately 1.0 Å seems “just right” for the selectivity filter.

Strikingly, the D→A mutant had the same permeability and conductivity sequences for monovalent cations as the wild-type pore (Fig. 5, B and E, and fig. S6). In addition, the wild-type and D→A mutant channels exhibited indistinguishable relative permeabilities among the ions tested (Fig. 5, D and E). These results suggest that the interaction distances between the selectivity filter of the channel and the monovalent cations moving through it are unperturbed by the D→A mutation. Thus, the D-locus is not a component of the selectivity filter that affects monovalent cation selectivity.

For the E→D mutant, by contrast, we observed a different monovalent permeability sequence, Li^+^ > K^+^ ~ Na^+^ > Cs^+^, in which K^+^ is similarly permeable to Na^+^ (Fig. 5, C and F, and fig. S6). This difference indicates a marked change in the field strength and an increased distance between the permeating ion and the open pore relative to wild type. Increased permeability of the larger K^+^ ion in the E→D mutant suggests, as the divalent cation experiments do, that the E→D mutant has a wider selectivity filter than the wild-type pore. The results of the monovalent selectivity experiments were consistent with the divalent selectivity ones: Mutation of the D-locus has very minor (if any) effects on ion selectivity, whereas mutation of the E-locus causes marked changes.

The primacy of the E-locus in the ion selectivity of the channel was unexpected. On the basis of structural studies of the channel and the sequence conservation of the D-locus, we and others had expected the D-locus to also have a substantial role in selectivity. Strong density ascribed to a Ca^2+^ ion has been observed within the E-locus in all x-ray or cryo-EM structures of MCU channels, suggesting that this region has an important role in selectivity (*21*–*29*, *59*). The structural studies also showed that the aspartic acid residues of the D-locus are exposed to the pore (Fig. 1B). From these observations, we and others proposed that a Ca^2+^ ion in the D-locus could interact with a Ca^2+^ ion in the E-locus and this ion-ion interaction would permit Ca^2+^ flux through the channel while providing high levels of selectivity (*24*, *25*). In support of this hypothesis, weaker density consistent with a second Ca^2+^ ion has been observed in the D-locus in some of the structures (*24*, *59*). The electrophysiological data presented here indicate that the ion selectivity properties of a channel completely devoid of the D-locus (the D→A mutant) are analogous to that of the wild-type channel. This indicates that the D-locus is not a primary component of the selectivity filter. On the other hand, the conservative E→D mutation of the E-locus markedly alters the ion selectivity properties of the channel. We find that the E-locus is responsible for two main features of Ca^2+^ selectivity in the Uniporter: a high-affinity block of monovalent currents by Ca^2+^ and the distinct divalent conductance sequence of the channel. We conclude that the E-locus serves as the selectivity filter of the channel and that the D-locus has a diminutive role in ion selectivity.

### Noise analysis identifies the E-locus as a high-throughput device

Considering our observations that the E-locus serves as the selectivity filter of the channel, we sought to address what mechanisms would allow for rapid ion conduction through it and what, if any, influence the D-locus has on the ion conduction rate. To address these questions, we investigated the single-channel properties of wild-type and mutant channels using nonstationary noise analysis. Using this technique, the amount of current flowing through a single channel (*i*) and the probability that the channel adopts an open conformation (*P*_O_) can be deduced (*60*). As shown in Fig. 1D, macroscopic Na^+^ currents, recorded from multiple channels under divalent-free conditions, displayed substantial fluctuations. These fluctuations or noise can be attributed to a collection of individual channels that switch between conductive (open) and nonconductive (closed) states in a stochastic manner. The fluctuations provided an experimentally tractable means to estimate the single-channel Na^+^ current (*i*_Na_). The magnitude of observed fluctuations (variance, σ^2^) is given by σ^2^ = *nP*_O_(1 − *P*_O_)*i*^2^. One can appreciate that the variance will be greatest when *P*_O_ = 0.5; in this case, each channel has a 50% probability of conducting ions as it stochastically switches between conductive and nonconductive states. On the other hand, when *P*_O_ is close to one or approaching zero, the variance will be minimized.

**Fig. 6.**
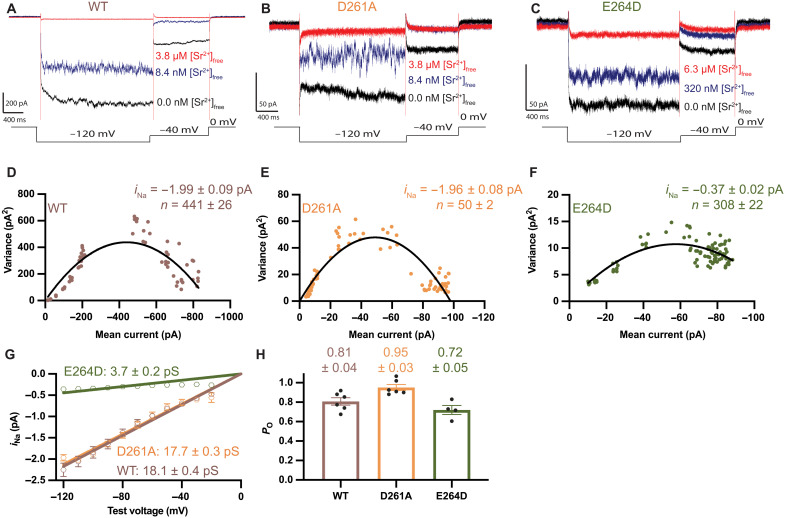
Noise analysis of Na^+^ currents to deduce single-channel properties. (**A** to **C**) Na^+^ currents through *Tc*MCU-EMRE and mutant channels exhibit substantial fluctuations (noise). The magnitude of the fluctuations depends on the extent of block by Sr^2+^. Representative current traces show that fluctuations observed for divalent-free conditions (black traces; without Sr^2+^) increase at an intermediate level of block by Sr^2+^ (blue) and decrease when higher concentrations of [Sr^2+^]_free_ are used (red). (**D** to **F**) Representative variance–versus–mean current relationships for *Tc*MCU-EMRE and mutant channels. [Sr^2+^]_free_ levels (from 2 nM to 6.3 μM) were used to vary the mean current. Solid lines represent fits of the data (Materials and Methods) to yield estimates for the single-channel sodium current (*i*_Na_) observed at −120 mV and the number of channels in the membrane (*n*). Errors given represent the error of the fit. (**G**) Single-channel conductance estimates. Noise analyses analogous to that in (D) to (F) were conducted for test voltages between −20 and −120 mV, from four or more separate bilayers for each protein construct. The deduced values of *i*_Na_ (with error bars representing the SEM) are plotted for each test voltage. The data can be well fit by linear regressions to yield the indicated single-channel conductances (errors represent errors in the fits). Near-linearity indicates that the single-channel conductance for Na^+^ does not vary substantially with the applied voltage. (**H**) *P*_O_ values for the protein constructs (at −120 mV) deduced from these experiments. Individual data points represent values deduced using different bilayers, with error bars representing the SEM.

To deduce *i*_Na_ using noise analysis, one must be able to modulate *P*_O_ (*60*). Borrowing from methods used to probe these properties for the Ca^2+^ channel Orai1 (*57*), we used the blockade of Na^+^ currents by Sr^2+^ to effectively modulate *P*_O_. Sr^2+^ was chosen for the blocking agent because its affinities for wild type and the D→A and E→D mutants were in concentration ranges that could be well controlled using a buffering system. [Sr^2+^]_free_ was titrated from 0 to 6.3 μM to vary the amount of block. The current traces (Fig. 6A) and a plot of σ^2^ versus mean current (⟨*I*⟩) (Fig. 6D) depict graphically that the variance observed in the absence of Sr^2+^ increases when ⟨*I*⟩ is reduced to approximately half its uninhibited value. On the other hand, when a higher concentration of Sr^2+^ is used to block most of the current, the variance is minimized. This parabolic relationship between σ^2^ and ⟨*I*⟩ was fit with the equation $\sigma^{2}=i_{Na}\langle I\rangle -\frac{{\left\langle I\right\rangle }^{2}}{n}$ to estimate *i*_Na_ (Fig. 6D) (*61*). Doing so for multiple recordings, we estimate that *i*_Na_ is −2.17 ± 0.05 pA at −120 mV, which corresponds to a single-channel conductance of 18.1 ± 0.4 pS (Fig. 6G). This value represents the average single-channel conductance, as the analysis does not discern whether the channel exhibits subconductance states. The result agrees with mitoplast recordings, in which the Uniporter exhibits evidence for multiple conductance states, with *i*_Na_ values at −120 mV between approximately −1.2 and −3.2 pA (*41*). Agreement with mitoplast recordings shows that the MCU-EMRE complex recapitulates the single-channel properties of the Uniporter without additional subunits. From the relationship between ⟨*I*⟩ and *P*_O_, ⟨*I*⟩ = *niP*_O_, we deduce that the unblocked channel exhibits a *P*_O_ of approximately 0.81 ± 0.04 at −120 mV when Na^+^ is the charge carrier (Fig. 6H).

We next evaluated *i*_Na_ for the mutants. Using the same approach as for wild-type protein, we find that the E→D mutation has a similar *P*_O_ to the wild-type channel (0.72±0.05) but that it has approximately fivefold lower *i*_Na_ (−0.44 ± 0.02 pA at −120 mV) and single-channel conductance (3.7 ± 0.2 pS) relative to it (Fig. 6, C, F, and G). The E→D mutation therefore exhibits both reduced ion selectivity and reduced single-channel conductance relative to the wild-type channel.

By contrast, the D→A mutation displayed single-channel properties similar to the wild-type channel: *i*_Na_ at −120 mV of −2.12 ± 0.04 pA, corresponding to a conductance of 17.7 ± 0.3 pS (Fig. 6, B, E, and G), and a *P*_O_ of 0.95 ± 0.03 (Fig. 6H). The observed *i*_Na_ at −120 mV corresponds to approximately 1.3 × 10^7^ Na^+^ ions flowing through an individual open channel per second. The results indicate that the E-locus operates as a high-throughput device that enables high conductivity.

### Noise analysis of Ca^2+^ currents

**Fig. 7.**
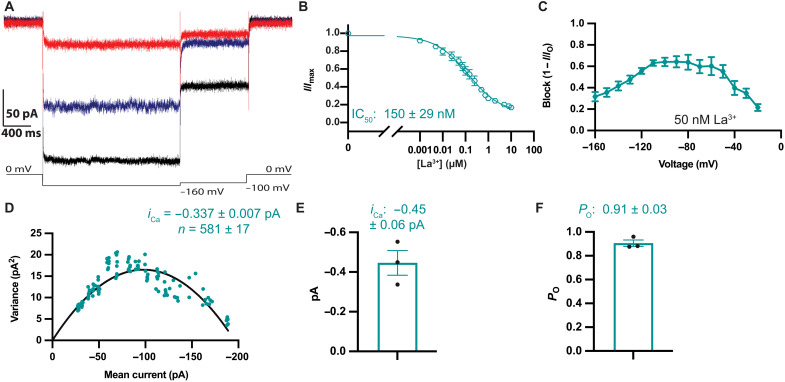
Noise analysis of Ca^2+^ currents. (**A**) Ca^2+^ currents through *Tc*MCU-EMRE are inhibited by La^3+^. Currents elicited in symmetric 40 mM Ca^2+^ are shown in the absence of La^3+^ (black) and in the presence of 50 nM (blue) or 10 μM (red) La^3+^ (on the trans side). Current fluctuations are apparent with 50 nM La^3+^. (**B**) Dose-response curve for the inhibition of Ca^2+^ currents by La^3+^ (40 mM symmetric Ca^2+^, at −160 mV). *I*_max_ is the mean uninhibited current at −160 mV. Hill coefficient is 0.7 ± 0.1. (**C**) The La^3+^ binding site is in the electric field. The extent of blockade of Ca^2+^ currents by 50 nM La^3+^ is shown as a function of the test voltage (calculated as in Fig. 3, G to I). (**D**) Representative variance–versus–mean current relationship for *Tc*MCU-EMRE. La^3+^ levels (from 1 nM to 10 μM) were used to vary the mean current. Solid line represents a fit of the data (Materials and Methods) to yield *i*_Ca_ (at −160 mV) and the number of channels (*n*). Error represents the error of the fit. (**E**) *i*_Ca_ deduced from noise analysis of three different bilayers (at −160 mV). (**F**) *Tc*MCU-EMRE exhibits high *P*_O_ at −160 mV with Ca^2+^ as the charge carrier. In (B) and (C), data points represent the mean of three recordings from different bilayers, with error bars representing the SEM. In (E) and (F), data points represent values derived from three different bilayers, with error bars representing the SEM.

Building off the work from noise analysis of Na^+^ currents, we next sought to evaluate the single-channel properties of Ca^2+^ conduction. Using an approach analogous to methods used to estimate *i*_Na_, we identified that lanthanum (La^3+^) is a potent blocker of Ca^2+^ currents through the channel. The measured apparent affinity of *Tc*MCU-EMRE for La^3+^, in the presence of 40 mM Ca^2+^, was 150 ± 29 nM (Fig. 7, A and B). With La^3+^ as a channel blocker, we used noise analysis of Ca^2+^ currents and estimated *i*_Ca_ = −0.45 ± 0.06 pA at −160 mV, which corresponds to a single-channel conductance of 2.8 ± 0.4 pS (Fig. 7, A, D, and E). This amount of current corresponds to approximately 1.4 × 10^6^ calcium ions flowing through the pore per second. We find that the unblocked channel exhibits a *P*_O_ value of approximately 0.91 ± 0.03 at −160 mV, with Ca^2+^ as the charge carrier (Fig. 7F). Unfortunately, macroscopic Ca^2+^ current levels were not sufficient to obtain reliable noise analysis data for the mutants.

We found that block of wild-type *Tc*MCU-EMRE by La^3+^ was voltage dependent (Fig. 7C). Blockade of Ca^2+^ currents by 50 nM La^3+^ increased with more negative voltages, reached a peak at approximately −80 mV, and declined at more negative voltages. The relief of block at the most negative voltages is consistent with the exit of La^3+^ into the cis chamber due to a punch-through effect. The voltage dependence of blockade indicates that the binding site for La^3+^ is located within the electric field. The different voltage-dependent pattern in comparison to Ca^2+^ blockade of Na^+^ currents, which displayed maximal block near neutral voltage, indicates that La^3+^ and Ca^2+^ bind at different positions within the channel at neutral voltage. We hypothesize that, similar to Ca^2+^, La^3+^ also interacts with the E-locus. Its voltage dependence suggests that its binding position at 0 mV is slightly closer to the IMS side of the E-locus than the high-affinity site for Ca^2+^.

### A multi-ion pore in the Uniporter: The E-locus can accommodate multiple Ca^2+^ ions

Having identified the E-locus as the primary selectivity filter and that it catalyzes rapid ion permeation, we suspected that the E-locus might be able to accommodate multiple Ca^2+^ ions despite the appearance of density interpreted as a single ion in the x-ray and cryo-EM structures. A classical model of selective ion permeation through an ion channel, for which there is ample electrophysiological evidence in K_V_Ca_V_ channels, and in glycine and γ-aminobutyric acid receptor chloride channels, involves multiple ions in a single file within the selectivity filter (*43*, *44*, *62*–*66*). X-ray structures of canonical tetrameric potassium channels show this clearly, with four distinct K^+^ sites within their selectivity filters, as first exemplified for the potassium channel KcsA (*67*, *68*) and subsequently for a mammalian K_V_ channel (*69*, *70*). Multiple ions in a single file, in conjunction with interactions between the permeating ions (ion-ion interactions), can allow both for selectivity among ions, which could be attributed to selectivity for ion binding, and for high throughput. However, the Uniporter, with a *K*_d_ of <2 nM for Ca^2+^, is distinct from other ion channels that have been characterized in that the affinity for the permeant ion is orders of magnitude tighter. The K^+^ channel KcsA, for example, has a *K*_d_ of approximately 0.5 mM for K^+^ (*43*, *71*). The Ca^2+^ channel Orai1, which is among the most selective metazoan Ca^2+^ channels known, has an affinity for Ca^2+^ of approximately 4 to 20 μM (*57*, *72*). However, the single-channel conductance of Orai1 (*i*_Ca_), estimated at 20 to 40 fS (*73*, *74*), is approximately three orders of magnitude lower than that of the Uniporter, despite the Uniporter’s more than 1000-fold greater affinity for Ca^2+^.

An electrophysiological hallmark of interactions between ions during permeation is the so-called anomalous mole fraction effect (AMFE) (*55*). If one considers a hypothetical ion channel containing multiple ions that interact with one another, the conduction through the channel could be affected by the ion-ion interactions. If the channel were able to conduct two different types of ions, Ca^2+^ and Ba^2+^, for example, then each would give rise to a certain conductance on their own. An AMFE can occur when a mixture of the ions is used in the experiment: The interactions between two different types of ions might result in a different conduction rate than would be expected from the average of the rates for each ion. This observed “anomalous” change in conduction is evidence for ion-ion interactions within the channel (*75*).

**Fig. 8.**
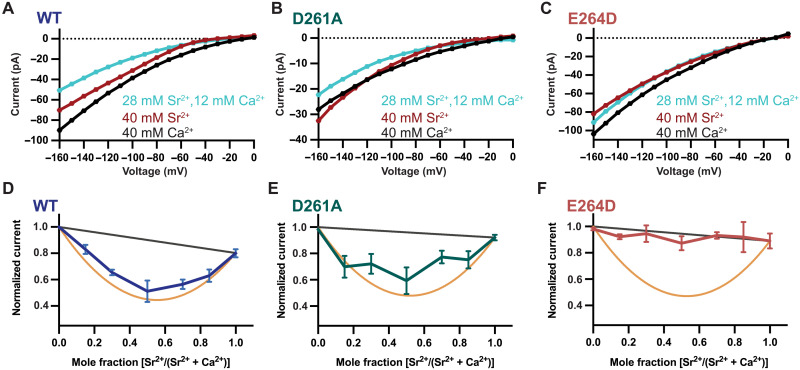
An AMFE indicates ion-ion interactions in the pore. (**A**) Representative *I*-*V* relationships for *Tc*MCU-EMRE reveal a substantial AMFE. *I*-*V* relationships for Ca^2+^ (black trace) or Sr^2+^ (red trace) alone or for a mixture of the ions (cyan trace) reveal that the mixture yields less current than either ion in isolation (indicating an AMFE). (**B** and **C**) Analogous *I*-*V* relationships for the D→A and E→D mutants. (**D** to **F**) Plots of mean currents observed at −160 mV as a function of Sr^2+^ mole fraction for *Tc*MCU-EMRE and mutant channels. The orange lines represent the theoretical minimal current (maximal AMFE) for the two-site model described in the text. The black lines represent theoretical data for the absence of an AMFE (with ions permeating independently from one another).

We used Ca^2+^ and Sr^2+^, which are similarly conductive through the channel, to look for an AMFE in *Tc*MCU-EMRE. The results were striking. Mixtures of Sr^2+^ and Ca^2+^ elicited a strong AMFE in the wild-type channel (Fig. 8, A and D). For example, an equal mixture of the ions yielded a nearly 50% reduction in current in comparison to either ion alone. These data provide evidence that selective ion conduction through the Uniporter operates through a multi-ion mechanism.

We used the D→A and E→D mutants to investigate the involvement of the D- and E-loci in multi-ion conduction. We found that the D→A mutation has negligible effect: This mutant displayed an AMFE that was analogous to the wild-type channel (Fig. 8, B and E). These results indicate that the D-locus is not responsible for the ion-ion interactions that underlie selective ion permeation through the channel. Although density that may represent a calcium ion is observed in the D-locus in some of the cryo-EM structures, this site is not involved in the ion-ion interactions that generate an AMFE for the wild-type channel.

On the other hand, an AMFE was not observed for the E→D mutant (Fig. 8, C and F). This indicates that the E-locus is involved in the multi-ion mechanism of selective permeation. Together, the AMFE experiments suggest that the E-locus itself can contain more than one Ca^2+^ ion. The geometrical constraints of the E-locus suggest that it could contain two Ca^2+^ ions (e.g., two Ca^2+^ ions could fit in a single file in the E-locus with appropriate spacing and coordination to the glutamate residues), but our data do not exclude the possibility of interactions with more than two Ca^2+^ ions. For example, molecular dynamics simulations suggest that three Ca^2+^ ions sometimes interact with a ring of acidic amino acids in the selectivity filter of the Ca^2+^-selective TRPV6 channel (*76*).

The AMFE that we observe is substantial. Typical effects in other ion channels result in less than 10% perturbation in ion conduction (*77*). The near 50% effect in the Uniporter suggests that the interactions between the permeating ions are particularly important for ion conduction. To provide context, we considered the following two-site scenario. An equal mixture of permeant ions A and B in a two-site pore would create four equally occupied possible ion configurations: A-A, A-B, B-A, and B-B. Channels containing A-A or B-B configurations would conduct ions at rates for the pure A or pure B cases, with the total rate through both as the average of these two rates. An AMFE could arise for channels containing A-B or B-A configurations. If those configurations conducted A or B ions at the same rates as for the pure A-A or B-B cases, then there would be no anomalous effect. On the other hand, if the A-B or B-A cases less (or more) efficiently conducted ions, then an AMFE would be observed. An extreme example of an AMFE would be if the A-B and B-A configurations were hardly conductive at all. In this case, the total conduction through the collection of channels would be reduced to approximately half of the mean conduction of the pure A and pure B cases. One can also imagine the effect in intermediate cases where the distribution between the four configurations was unequal, for example, when the concentration of A used in the experiment was higher than that of B. The theoretical maximum AMFE for this two-site model for Ca^2+^ and Sr^2+^ in *Tc*MCU-EMRE is plotted in Fig. 8 (D to F, orange lines). The absence of an AMFE is plotted as a black line. Comparison with the data indicates that the AMFE is approximately 90% of the maximum possible value. This suggests that the A-B and B-A configurations (e.g., Ca^2+^-Sr^2+^ and Sr^2+^-Ca^2+^) collectively conduct ions at approximately 10% of the rate observed for Ca^2+^ or Sr^2+^ alone. The magnitude of the AMFE for the channel is among the greatest observed for an ion channel and indicates that strong energetic coupling between ions underlies selective ion permeation in the Uniporter. Our mutational studies indicate that this ion-ion coupling does not depend on the D-locus and indicate that it occurs in the E-locus.

### Low-affinity interaction of multiple Ca^2+^ ions in the filter enables Ca^2+^ conduction

The electrophysiological studies presented here indicate that the E-locus constitutes the selectivity filter of the Uniporter and that it forms a high-affinity binding site for Ca^2+^ (*K*_d_ < 2 nM). Cryo-EM densities from structures of the Uniporter are consistent with a single Ca^2+^ ion in the E-locus, but our AMFE experiments indicate that multiple Ca^2+^ ions can be accommodated in it. We sought to reconcile these observations. As observed previously (*1*, *33*, *41*), while Na^+^ conduction through the channel is blocked by nanomolar levels of calcium, Ca^2+^ does not effectively permeate through the channel until millimolar levels are reached. These drastically different apparent affinities are another suggestion that multiple Ca^2+^ ions can interact with the pore.

**Fig. 9.**
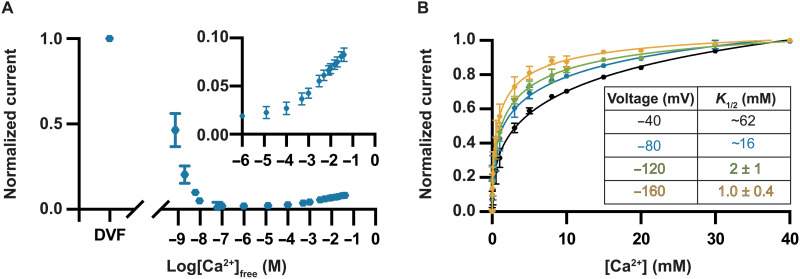
Ca^2+^ saturation. (**A**) Na^+^ currents through *Tc*MCU-EMRE are inhibited by nanomolar levels of Ca^2+^, but Ca^2+^ does not appreciably conduct until millimolar levels are reached. Current level starting from the DVF condition (and normalized to it) is plotted versus [Ca^2+^]_free_. Inset shows Ca^2+^ conduction in the upper range of [Ca^2+^]_free_. Data are from test voltages of −80 mV. (**B**) Ca^2+^ saturation is strongly voltage dependent. Normalized current versus [Ca^2+^] is plotted for the indicated test voltages. Solid lines represent fits of the data as described in Materials and Methods. The apparent *K*_1/2_ varies from approximately 62 mM at −40 mV to 1 mM at −160 mV. Currents are normalized to the values observed using 40 mM Ca^2+^ for each of the test voltages. Errors given in the table are errors of the fit. *K*_1/2_ values for −40 and −80 mV are approximate because the currents did not fully saturate using 40 mM CaCl_2_. Error bars at given concentrations represent the SEM from three independent experiments using separate bilayers.

To better understand the loading of multiple ions in the filter, we evaluated the Ca^2+^ saturation activity of the wild-type protein and its dependence on voltage. By titrating the amount of Ca^2+^ between 1 μM and 40 mM, we found that the wild-type protein displayed increasing Ca^2+^ currents as the concentration was increased (Fig. 9A). These currents could be fit by a standard saturation curve with a *K*_1/2_ of approximately 16 mM when the applied voltage was −80 mV (Fig. 9B). We found that *K*_1/2_ was strongly dependent on the applied voltage, ranging from approximately 62 mM at −40 mV to only 1 mM at −160 mV (Fig. 9B). The data indicate that the loading of multiple ions is concentration dependent and facilitated by voltage. The negative electric potential of the mitochondrial matrix would increase the likelihood of loading multiple Ca^2+^ ions. The results suggest that the presence of multiple Ca^2+^ ions in the E-locus underlies the high Ca^2+^ conductivity of the Uniporter.

## DISCUSSION

When purified and reconstituted into planar lipid membranes, the *Tc*MCU-EMRE complex recapitulatedelectrophysiological properties of the endogenous Uniporter, including its high selectivity for Ca^2+^ and single-channel conductance (*1*, *41*). The reconstituted system afforded the opportunity to study the electrical properties of mutations within the D- and E-loci of the pore. From structures showing that both loci are exposed to the pore, it has been hypothesized that both loci function in concert as the selectivity filter of the channel (*24*, *25*). Unexpectedly, the electrophysiological data presented here indicate that the E-locus constitutes the selectivity filter and that the D-locus has diminutive influence on selectivity. We find that E-locus forms the high-affinity site for Ca^2+^ that blocks Na^+^ currents. The E-locus can interact with multiple Ca^2+^ ions, as evidenced by a robust AMFE, and it becomes highly conductive when multiple Ca^2+^ ions are within it. The loading of multiple Ca^2+^ ions within it occurs in a voltage-dependent manner and may account for the voltage dependence of the Uniporter.

Structures of *Tc*MCU-EMRE reveal the conformation of the E-locus (*22*, *29*). This conformation is indistinguishable from that in all other MCU structures, including human MCU-EMRE complexes (*21*–*29*, *59*). In the structures, the four glutamate residues of the E-locus coordinate a bound Ca^2+^ ion directly. The glutamate residues of the E-locus are constrained by the architecture of the surrounding region. The structural integrity of the E-locus is bolstered by packing with the neighboring tryptophan and proline residues and by hydrogen bonds that are made to the nitrogen atom of the tryptophan (fig. S1) (*21*–*29*, *59*). The tight packing in this region suggests that glutamate carboxylate moiety is unlikely to adopt different rotamer conformations. A highly structured, preformed binding site would tend to have higher affinity for an ion in comparison to one that is more plastic through entropic terms in the free energy (*78*).

In the superfamily of cation channels of which Na_V_ channels are the founding member, which include K_V_, Ca_V_, and TRP channels and others, the selectivity filters are formed from loop regions (*67*, *70*, *79*–*82*). In some of these channels, the selectivity filter adopts different conformations under certain circumstances. For example, the selectivity filter of the K^+^ channel KcsA adopts conductive or collapsed conformations, depending on the concentration of K^+^ (*79*, *83*). Ion selectivity filter conformational changes also underlie pH dependence and inactivation gating in certain K2P and Shaker potassium channels (*84*, *85*). The observations that the selectivity filter of MCU has the same conformation in all structures determined to date, including those with MICU1-MICU2 bound, suggest that the selectivity filter does not undergo large conformational changes. The α-helical nature of the filter may provide stability to the protein backbone and limit the side-chain conformations of the glutamate residues.

Each glutamate residue of the E-locus is positioned such that its two oxygen atoms line the ion conduction pore with one closer to the IMS side and one closer to the matrix side (Fig. 1B). In the context of the tetrameric MCU assembly, this creates two rings, each containing four oxygen atoms within the E-locus: a lower ring closer to the IMS side and an upper ring closer to the matrix side (Fig. 1B). In the structures, which represent the channel at 0 mV and in low concentrations of Ca^2+^ (between ~2 nM and 2 mM) relative to the *K*_1/2_ of saturation, density consistent with a single calcium ion is positioned between the two rings, where it would be directly coordinated by all four glutamate residues (Fig. 1B) (*21*–*29*, *86*). The lower ring of oxygen atoms has narrower diameter than the upper one in the atomic coordinates, which could suggest a stronger interaction between Ca^2+^ and the lower ring (Fig. 1B). However, the precise locations of the Ca^2+^ ion and the coordinating oxygens (and the possibility that the ion density could represent two ions) are limited by the resolutions of the structures, which are determined at up to 3.1 Å resolution and have coordinate errors of 1.5 Å or more. Nevertheless, the data presented in this work indicate that the density for a Ca^2+^ ion within the E-locus represents the high-affinity site that blocks monovalent current through the channel. The observation that blockade is voltage dependent indicates that this site is located within the electric field. We find that the E→D mutation, which would increase the diameter of the pore in this region by approximately 2 Å, produces a channel with lower ion selectivity for Ca^2+^. Furthermore, we observe that the conductivity ratios for monovalent and divalent cations in the wild-type pore are correlated with ionic radii. The results suggest that the dimensions of the selectivity filter formed by the E-locus are crucial for Ca^2+^ selectivity. More precisely defined interatomic distances garnered by higher-resolution structures would provide further insight.

The approximately 10-Å-wide dimensions of the pore at the D-locus revealed by the structures (measured between oxygen atoms across the pore) indicate that interaction with Ca^2+^ at this position could be water-mediated (Fig. 1B). Unlike the E-locus, the side chains of the D-locus do not make interactions with nearby residues, and therefore, their conformations are more flexible. Accordingly, the aspartate side chains of the D-locus have weaker density in the x-ray and cryo-EM structures than the glutamate side chains of the E-locus (*21*–*29*, *59*). The studies presented here indicate that the D-locus has very minor influence on ion selectivity. Removal of the acidic charge at this locus with the D→A mutation creates a channel that is highly similar in its electrical properties to the wild-type MCU-EMRE complex. These properties include a high-affinity block of Na^+^ currents by Ca^2+^ and the same ion Ca^2+^ ~ Sr^2+^ >> Mn^2+^ ~ Ba^2+^ > Mg^2+^ conductivity sequence as the wild-type channel. The D→A mutant also exhibited an AMFE, indicating that ion conduction through the mutant channel involves ion-ion interactions. Solvent-exposed acidic residues such as the D-locus near the mouths of cation channels have been proposed to concentrate cations in their vicinity (*87*). Although the D-locus may have such an effect, we find that *i*_Na_ values for the wild-type and D→A mutant channels are analogous, which suggests that the D-locus does not have a major influence on the ion conduction rate, at least for Na^+^. Further experiments are needed to evaluate whether the E-locus influences the Ca^2+^ conduction rate.

Although it has very little role in Ca^2+^ selectivity or *i*_Na_, the D-locus is highly conserved among MCU channels. A possible explanation for this conservation may be the interaction of the D-locus with MICU regulatory complexes (*21*, *22*). In metazoan organisms, heterodimers of MICU1 and MICU2 bind to the D-locus when the concentration of Ca^2+^ in the IMS is at a low resting level (below approximately 1 μM) (*21*–*23*). When cytosolic Ca^2+^ levels rise, a conformational change in MICU1-MICU2 releases its interaction with the D-locus, thereby allowing Ca^2+^ flux through the pore and helping to restrict the permeation of Ca^2+^ to activation events (*21*–*23*, *53*, *88*). Regulation of MCU-EMRE by MICU1-MICU2 may impart a degree of selectivity to the channel in a physiological setting in the sense that, at submicromolar concentrations of Ca^2+^, MICU1-MICU2 would reduce the possibility of K^+^ or other cation permeation through the pore. Even under submicromolar [Ca^2+^] conditions, cryo-EM structures of MICU1-MICU2 in complex with MCU-EMRE indicate that Ca^2+^ occupies the blocking site in the E-locus (*21*, *22*). In a manner of speaking, the channel is doubly blocked at resting concentrations of Ca^2+^ by MICU1-MICU2 and by Ca^2+^.

That the Uniporter achieves exquisite selectivity for Ca^2+^ and yet can conduct approximately 10^6^ calcium ions per second is an impressive feat when one considers the high affinity (*K*_d_ < 2 nM) with which Ca^2+^ blocks monovalent current. *K*_d_ is related to the off-rate (*k*_off_) and the on-rate (*k*_on_) by the following relationship: *K*_d_ = *k*_off_/*k*_on_. If one considers a hypothetical hemispherical binding site that acts as a sink for ions at the mouth of the pore, then the fastest *k*_on_ from bulk solution is limited by diffusion and is approximately 2 × 10^9^ M^−1^ s^−1^ (*55*). If the *K*_d_ of this binding site is 2 nM, then the *k*_off_ for that site is approximately 0.1 s^−1^; the site would allow the release (conduction) of one ion approximately every 10 s. Even the fastest-releasing 2 nM affinity binding site is approximately 10 million times slower than the observed rate of ~10^6^ ions/s at which Ca^2+^ ions can flow through the Uniporter. The Uniporter is an extreme example among highly selective ion channels that ion binding affinity alone cannot underlie selective ion conduction at the observed rates.

From observations as early as Hodgkin and Keynes (*89*) made for K^+^ channels or by Eisenman *et al.* (*90*) relating to the behavior of thin glass films, it was proposed that ion channels can contain multiple interacting ions in the pore. From subsequent work on voltage-gated Ca^2+^ channels (*44*, *77*, *91*), the multi–ion pore hypothesis was proposed as a mechanism for the selective permeation of ions at rates that could reach those observed experimentally. Multiple interacting ions in a single file could account for selective ion conduction because one ion could replace the binding of the next, and thus, the pore would not be devoid of ions even as they permeate. The Uniporter is an extraordinary example of this. The multi-ion pore in the Uniporter elevates the rate of ion conduction from approximately 1 ion every 10 s for a single site to the observed rate of approximately 10^6^ ions per second. We find that the E-locus is responsible for this feat, which it achieves it by facilitating the interaction of multiple Ca^2+^ ions with one another.

Although our experiments do not exclude the possibility that more than two Ca^2+^ ions could be present, the dimensions of the E-locus suggest that two Ca^2+^ ions could fit comfortably within the ring of glutamate residues. The data suggest that the presence of more than one Ca^2+^ ion in the E-locus is both concentration and voltage dependent. For example, at 100 nM [Ca^2+^] and 0 mV, only one Ca^2+^ ion would be bound on average. This ion would occupy the blocking site in the E-locus, as exemplified in the available x-ray and cryo-EM structures. At 10 mM [Ca^2+^] and −160 mV, our results suggest that two (or possibly more) Ca^2+^ ions would be present within the E-locus.

We find that La^3+^ potently blocks Ca^2+^ current through *Tc*MCU-EMRE. On the basis of its high-affinity interaction, its similar ionic radius (1.05 Å) to that of Ca^2+^ (1.00 Å), and that lanthanides (La^3+^ or Gd^3+^) often bind approximately where calcium ions do in the selectivity filters of Ca^2+^ channels (*92*–*96*), we hypothesize that La^3+^ binds in the E-locus and competes for Ca^2+^. Its binding there would suggest that La^3+^ binding is preferential to the presence of Ca^2+^ ions within the E-locus. It would also suggest that the E-locus can accommodate a charge of +3 and that a charge of +2 (one Ca^2+^ ion) or +4 (two Ca^2+^ ions) is less favorable. This situation is reminiscent of the selectivity filter of canonical potassium channels, exemplified in studies of KcsA, which suggest that the filter accommodates more than two, but less than three, K^+^ ions on average (*68*, *79*). This metastable condition is thought to contribute to selective ion permeation in those channels, as the selectivity filter toggles between containing two and three K^+^ ions. We hypothesize that a similar situation is at work in the E-locus; it may alternate between interacting with one and two Ca^2+^ ions during conduction.

**Fig. 10.**
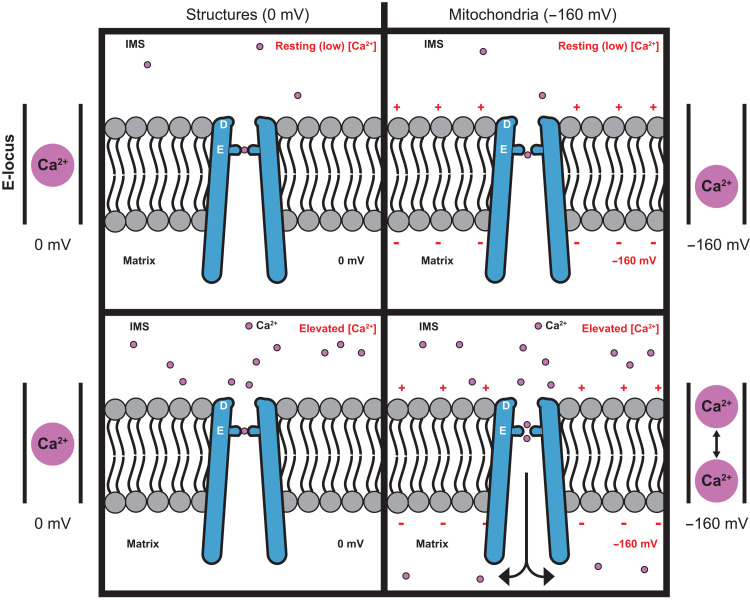
Ca^2+^ selectivity mechanism. Schematic of the proposed mechanism for Ca^2+^ selectivity and Ca^2+^ throughput described in the main text is shown. The ion pore is represented by a blue shape. The D- and E-loci are denoted with white lettering. The E-locus is also represented by vertical lines to the left and right of the main panels. At low resting levels of Ca^2+^, a Ca^2+^ ion occupies the high-affinity site within the E-locus (top). The binding of this ion blocks monovalent cation permeation. Because the E-locus is located within the electric field, the negative voltage of the matrix relative to the cytosol causes a slight downward displacement of the blocking Ca^2+^ ion (top right) relative to its position at neutral voltage (top left). At elevated levels of Ca^2+^, two (or possibly more) Ca^2+^ ions can be accommodated in the E-locus (bottom right). The presence of multiple ions in the E-locus is facilitated by the electric field. Mutual repulsion between the ions facilitates dissociation of an ion. The negative voltage of the matrix biases the release of the lower Ca^2+^ ion, which can flow through the remainder of the pore (curved arrows, bottom right). Currently available x-ray and cryo-EM structures of MCU/MCU-EMRE complexes represent neutral voltage conditions with subsaturating concentrations of Ca^2+^ (left) in which a single Ca^2+^ is bound in the E-locus.

We propose a model for selective Ca^2+^ conduction by the Uniporter shown in Fig. 10. At a low concentration of Ca^2+^ and the absence of a voltage gradient across the membrane, a single Ca^2+^ ion would be bound within the E-locus. This ion, located approximately between the two rings of oxygen atoms of the glutamate residues, represents the high-affinity Ca^2+^ site that blocks Na^+^ current and the configuration observed in the structures of the Uniporter to date. Our data indicate that the blocking Ca^2+^ ion is located within the voltage field across the membrane because the *K*_d_ of block depends on the voltage applied. If the voltage were increased, for example, to typical levels for mitochondria, approximately −160 mV, the electric field would exert a downward force (toward the matrix) on the Ca^2+^ ion. The force would cause displacement of the Ca^2+^ ion toward the matrix, where it would be closer to the lower ring of oxygen atoms than the upper ring. If the field were strong enough, the force on the Ca^2+^ ion could cause it to be pulled from the E-locus (a punch-through effect). Evidence that punch-through occurs is that the extent of block for a given concentration of Ca^2+^ decreases as the voltage is increased to more negative values. In the model, downward displacement of the Ca^2+^ would create space for a second ion to bind, presumably where it would interact with the upper ring of oxygen atoms. We have shown that the apparent affinity for loading of multiple Ca^2+^ ions into the E-locus is approximately seven orders of magnitude lower than the *K*_d_ for the blocking Ca^2+^. The loading of multiple ions is strongly dependent on the applied voltage such that at −40 mV, *K*_1/2_ is approximately 62 mM, whereas at the typical mitochondrial voltage of −160 mV, *K*_1/2_ is approximately 1 mM. The average positions of the Ca^2+^ ions in the E-locus would be due to a combination of the attractive forces with the coordinating oxygens, electrostatic forces due to the membrane voltage, and repulsive forces between the ions. When two Ca^2+^ ions are present within the E-locus, we hypothesize that neither ion would occupy the blocking site. Rather, both ions would flank the blocking site, with one above and one below it. A negative voltage would bias the release of the lower Ca^2+^ into the remainder of the pore, thus resulting in Ca^2+^ conduction into the matrix. We suspect that the apparent *K*_1/2_ that we measure for Ca^2+^ saturation represents the overall affinity of multiple ions for the conductive configuration.

Continuing with the model, if access to the E-locus is limited by diffusion, then the maximum on-rate of the second ion (*k*_on_) would be approximately 2 × 10^9^ M^−1^ s^−1^ (*55*). With an apparent dissociation constant of 1 mM (the *K*_1/2_ value at −160 mV), the off-rate of one of the two ions (the ion conduction rate) would be approximately 2 × 10^6^ s^−1^. This value is satisfyingly consistent with the observed conduction rate of approximately 1.4 × 10^6^ calcium ions per second (at −160 mV and saturating Ca^2+^) from the noise analysis experiments. In the context of the negative mitochondrial potential (approximately −160 mV) applying a downward force, the lower ion would be more likely to dissociate. With the release of the lower Ca^2+^ ion, the upper Ca^2+^ ion would be drawn toward the blocking site by coordination with the glutamate oxygens (and also pulled downward by the electric field), returning the situation to the original configuration and priming the E-locus for interaction with another Ca^2+^ ion. If one considers the maximal ion throughput rate of the channel (approximately 10^6^ per ion per second) in comparison to an estimated rate of diffusion to the mouth of the pore of 2 × 10^9^ M^−1^ s^−1^ and that ion conduction occurs as a result of a multi-ion pore, then it becomes apparent that with sufficient [Ca^2+^] for conduction (~1 mM at −160 mV), the rate for filling by the second ion is similar to the rate of release. This suggests that the presence of two ions is transient: As soon as the second ion arrives, the first one leaves.

This study identifies that the E-locus is the selectivity filter of the Uniporter. Through a multi-ion mechanism, the E-locus imparts the channel with both an exquisite selectivity for Ca^2+^ and the ability to conduct ions rapidly. The physiochemical environment of the E-locus and its interactions with Ca^2+^ ions are tuned both to prevent ion leakage at low concentrations of Ca^2+^ that would otherwise disrupt the mitochondrial voltage gradient and to permit Ca^2+^ permeation when cytosolic levels rise. Through tightly and selectively controlling Ca^2+^ influx, the efficient design of the Uniporter communicates cellular energy needs to mitochondria to fulfill these demands.

## MATERIALS AND METHODS

### Protein purification

The *Tc*MCU-EMRE expression construct previously referred to as *Tc*MCU-EMRE_EM2_ (*29*) was used for functional studies in this work. Mutations were made using the New England Biolabs Q5 site-directed mutagenesis system, and coding regions were fully sequenced. *Tc*MCU-EMRE and mutant proteins were expressed as fusion proteins with the Venus variant of green fluorescent protein (GFP) and purified as described previously (*29*), with minor modifications. The following mutations were made and evaluated for protein stability [e.g., using fluorescence-detection size-exclusion chromatography (*97*) and protein purification]: D261A, E264D, E264Q, E264A, Y268F, Y268A, and T271A. Among these, D261A and E264D yielded the protein that could be purified. Plasmids were transiently transfected into Expi293 cells (Invitrogen). One milligram of plasmid was combined with 3 mg of PEI25k in 100 ml of Opti-MEM media (Invitrogen) and incubated at room temperature for 20 min. The plasmid-PEI25k mixture was then added to 900 ml of Expi293 cells (3.0 × 10^6^ to 3.5 × 10^6^ cells/ml; in Expi293 expression media), and the cells were incubated at 37°C for 16 hours, after which point 10 mM sodium butyrate (Sigma-Aldrich) was added and the cells were cultured at 30°C for an additional 48 hours. Cells were harvested by centrifugation (800*g* at 4°C for 20 min), washed once with phosphate-buffered saline, flash-frozen in liquid nitrogen, and stored at −80°C until use for protein purification.

Crude cell membranes were prepared in the following manner. All steps were performed at 4°C unless otherwise noted. The cell pellet from 1 liter of culture was thawed in a bath of cool water. The pellet was then resuspended in 100 ml of lysis buffer [100 mM Hepes-NMDG (*N*-methyl-d-glucamine) (pH 7.6), 200 mM NaCl, deoxyribonuclease I (0.1 mg/ml), leupeptin (1.5 μg/ml), pepstatin A (1.5 μg/ml), 1 mM 4-benzenesulfonyl fluoride hydrochloride, 1 mM benzamidine, 1 mM phenylmethylsulfonyl fluoride (PMSF), and 1:500 dilution of aprotinin; Sigma-Aldrich, catalog number A6279] and lysed by sonication (15% power, 5 s on and 10 s off, 5 min in total; Branson SFX 250). The crude membrane fraction was pelleted by centrifugation (70,000*g* for 1 hour), resuspended in 30 ml of resuspension buffer [100 mM Hepes-NMDG (pH 7.5), 200 mM NaCl, 20% glycerol, and 1:500 dilution PI mix III; EMD Millipore], flash-frozen using liquid nitrogen, and stored at −80°C until further use.

Cardiolipin (18:1; Avanti; dried from a chloroform solution under argon gas, washed with pentane, and desiccated at room temperature overnight while shielded from light) was resuspended to 10 mg/ml in water via end-over-end mixing at room temperature. After 1 hour, the lipids were sonicated thoroughly using a water bath, 2% (w/v) *n*-dodecyl-β-d-maltopyranoside (DDM) powder (Anatrace) was added, and the sample was mixed end-over-end for an additional hour at room temperature. The DDM-solubilized cardiolipin mixture was subsequently stored on ice in the dark until use.

Once the membranes were thawed (using a bath of cool water), final concentrations of 1 mM PMSF (100 mM stock in ethanol), 0.1 mg/ml cardiolipin that had been solubilized with DDM (preparation described above), and 1% (w/v) DDM (from powder) were added, and the sample was stirred for 1 hour to extract the membrane proteins. Detergent-solubilized proteins were separated from the insoluble fraction by centrifugation (70,000*g* for 30 min). The supernatant was filtered through a 0.22-μm polystyrene membrane (Millipore Sigma, catalog number S2GPU01RE). GFP nanobody resin (2 ml) (*98*) was added, and the sample was rotated for 1 hour for binding. The resin was then transferred to a gravity column and washed with 70 ml of wash buffer 1 [20 mM Hepes-NMDG (pH 7.6), 150 mM NaCl, 1 mM DDM, and 0.01 mg/ml cardiolipin], 50 ml of wash buffer 2 [20 mM Hepes-NMDG (pH 7.6), 500 mM NaCl, 1 mM DDM, and 0.01 mg/ml cardiolipin], and then 20 ml of wash buffer 1. *Tc*MCU-EMRE was liberated from the affinity column by proteolysis with 0.1 mg of PreScission protease (3 hours of incubation in buffer supplemented with 1 mM dithiothreitol). The eluted protein was concentrated to approximately 0.5 ml using a 100-kDa MWCO concentrator (Amicon Ultra-15) and applied to a Superose 6 Increase 10/300 GL size exclusion column (GE Healthcare) that was equilibrated in 20 mM Hepes-NMDG (pH 7.6), 150 mM NaCl, 1 mM DDM, and 0.01 mg/ml cardiolipin. Peak fractions containing *Tc*MCU-EMRE were evaluated for purity by SDS–polyacrylamide gel electrophoresis and used for liposome reconstitution.

### Reconstitution of *Tc*MCU-EMRE into liposomes

All lipids were obtained from Anatrace. A mixture of lipids was prepared by combining chloroform solutions containing 30 mg of 1-palmitoyl-2-oleoyl-*sn*-glycero-3-phosphoethanolamine (POPE), 10 mg of 1-palmitoyl-2-oleoyl-*sn*-glycero-3-phosphoglycerol (POPG), and 3 mg of cardiolipin (18:1) in a glass tube; evaporating the chloroform using argon; dissolving the lipids in pentane; drying the lipids under argon; and placing the lipids under vacuum overnight while protecting them from light. Reconstitution buffer [20 mM Hepes-NMDG (pH 7.6) and 450 mM NaCl] was added to the dried lipids to yield a total lipid concentration of 20 mg/ml. The lipids were incubated with end-over-end mixing for 1 hour, and the mixture was sonicated in a water bath for a total of 5 min in 1-min on/off intervals. The lipids were subsequently solubilized by adding 2% DDM (w/v) from powder. Following an approximately 1-hour incubation (with end-over-end mixing at room temperature), this DDM-solubilized PE:PG:cardiolipin mixture was used immediately for reconstitution.

For reconstitution into liposomes, freshly prepared protein was concentrated to ~100 μl at ~1 mg/ml using a 100-kDa MWCO concentrator (Amicon Ultra-2) and mixed with the DDM-solubilized PE:PG:cardiolipin lipids to yield a 1:20 protein-to-lipid ratio (for example, 100 μg of protein would be mixed with 2 mg of PE:PG:cardiolipin). After mixing end-over-end overnight at 4°C, an equal volume of 100 mM methyl-β cyclodextrin (MBCD; Sigma-Aldrich; dissolved in reconstitution buffer) was added every 8 hours for 24 hours to remove detergent (*99*). For example, a 100-μl sample was supplemented with three additions of 100 μl of MBCD every 8 hours to form proteoliposomes. The proteoliposomes were then collected by centrifugation (194,000*g*, 30 min, 4°C). After discarding the supernatant, the proteoliposomes were resuspended in reconstitution buffer [20 mM Hepes-NMDG (pH 7.6) and 450 mM NaCl] to yield an approximate lipid concentration of 20 mg/ml, sonicated briefly, flash-frozen using liquid nitrogen in 20-μl aliquots, and stored at −80°C until use in bilayer recordings.

### Planar lipid bilayer electrophysiology

Generally following the procedures previously described (*100*), frozen liposomes were thawed and sonicated for ∼10 s using a water bath (Ultrasonic Cleaner, Laboratory Supplies Company). All data are from recordings made using the Warner planar lipid bilayer workstation (Warner Instruments). Two aqueous chambers (4 ml) were filled with bath solutions. Chlorided silver (Ag/AgCl) wires were used as electrodes, submerged in 3 M KCl, and connected to the bath solutions via agar-KCl salt bridges [2% (w/v) agar and 3 M KCl]. The bath solutions were separated by a polystyrene partition with an approximately 200-μm hole across which a bilayer was painted using POPE:POPG in *n*-decane [3:1 (w/w) ratio at 20 mg/ml]. Liposomes were fused under an osmotic gradient across the bilayer (*34*), under divalent-free conditions, with solutions consisting of 150 mM NaCl (cis side) or 15 mM NaCl (trans side), 1 mM EGTA-NMDG, 1 mM EDTA-NMDG, and 20 mM Hepes-NMDG (pH 7.6). Liposomes were added, 1 μl at a time, to the cis chamber to a preformed bilayer until currents were observed. We found that divalent-free conditions yielded better fusion with the bilayer than divalent-containing solutions. Solutions were then made symmetrical by adding 135 mM NaCl (from a 5 M NaCl stock) to the trans side. Unless noted, all reagents were purchased from Sigma-Aldrich. All electrical recordings were taken at room temperature (22° to 24°C) and represent the mean current at a given test voltage (calculated from the 0.8- to 2.5-s window of the voltage protocol). Detailed buffer compositions for electrophysiological experiments are given in tables S1 to S9.

Currents were recorded using an Axopatch 200B amplifier (Axon Instruments), filtered at 1 kHz, and digitized at 5 kHz using the Clampex 10.4 program (Axon Instruments). Data were analyzed using Clampfit 10.4 (Axon Instruments). In all cases, currents from bilayers without channels are subtracted. Error bars represent the SEM of at least three experiments, each using a different bilayer. We define the side to which the vesicles are added as the cis side and the opposite trans side as electrical ground, so that the transmembrane voltage is reported as *V*_cis_ − *V*_trans_.

For experiments aimed at studying the channel properties of monovalent cations, the trans chamber was perfused with approximately 35 ml of a solution containing 150 mM test cation (K^+^, Li^+^, and Cs^+^) as a chloride salt under divalent-free conditions [1 mM EGTA-NMDG, 1 mM EDTA-NMDG, and 20 mM Hepes-NMDG (pH 7.6)]. The voltage protocols are outlined in the figure legends.

The Goldman-Hodgkin-Katz equation was solved to determine permeability ratios between monovalent cations under bi-ionic conditions, where *E*_rev_ is the measured reversal potential, *X* is the permeant cation perfused to the trans compartment, and sodium is in the cis compartment. The equation takes the following form

$$E_{\mathrm{rev}}=\frac{\mathrm{RT}}{zF}\ln\frac{P_{x}[X^{+}]}{P_{\mathrm{Na}}[{\mathrm{Na}}^{+}]}$$ (1)

To record Ca^2+^ currents, channels were first fused with the bilayer as described above to measure Na^+^ currents under divalent-free conditions. For each experiment, we confirmed that the addition of approximately 1 μM [Ca^2+^]_free_ inhibited the Na^+^ currents. To measure Ca^2+^ currents, CaCl_2_ (typically 40 mM) was subsequently added to both chambers. For experiments aimed at studying the channel properties of different divalent cations, the trans chamber was then perfused with approximately 35 ml of a solution [20 mM Hepes-NMDG, 1 mM EGTA-NMDG, and 1 mM EDTA-NMDG (pH 7.6)] containing 40 mM test cation (Sr^2+^, Ba^2+^, Mn^2+^, or Mg^2+^) as a chloride salt. We chose not to estimate relative permeability ratios among divalent cations because the high selectivity for Ca^2+^ makes reversal potentials difficult to estimate (the *I*-*V* relationships asymptote along the voltage axis). Relative conductances (γ*_X_*/γ_Ca_) were determined by dividing the mean current at −160 mV for a given test ion by the mean current observed at −160 mV for Ca^2+^ from the same membrane.

For experiments using RuRed (Sigma-Aldrich), 1 μM RuRed was added to the cis chamber to inhibit channels with their IMS side facing that chamber. To assess the binding affinity, RuRed was then titrated into the trans chamber from 2 nM to 1 μM. For final concentrations less than 10 nM, a 2 μM stock of RuRed in dimethyl sulfoxide (DMSO) was used; for final concentrations up to 1 μM, a 200 μM stock in DMSO was used. Currents were normalized by dividing by the maximum current recorded at −160 mV in the absence of RuRed. The dose response was fit in GraphPad Prism 9 to the “log(inhibitor) vs. normalized response -- variable slope” equation, in which *X* represents the concentration of RuRed

$$\frac{I}{I_{\max}}=\frac{1}{\left\{1+10^{\left[({\mathrm{logIC}}_{50}-X)\times h\right]}\right\}}$$ (2)

To assess the ability of Ca^2+^ or Sr^2+^ to inhibit Na^+^ currents, CaCl_2_ or SrCl_2_ was added into standard divalent-free bath solutions to obtain the desired concentrations of [Ca^2+^]_free_ and [Sr^2+^]_free_, calculated using MaxChelator (https://somapp.ucdmc.ucdavis.edu/pharmacology/bers/maxchelator/webmaxc/webmaxcE.htm). A range of [Ca^2+^]_free_ and [Sr^2+^]_free_ from approximately 2 nM to 6.3 μM was selected, appreciating that 2 nM is the lower limit of chelation for EDTA and EGTA. The dose response was fit in GraphPad Prism 9 to Eq. 2, with *X* representing the concentration of the divalent cation.

Ca^2+^ saturation experiments were performed by varying [Ca^2+^]_free_ from 1 μM to 40 mM using CaCl_2_. The dose-response data were fit to the equation “specific binding with hill slope,” as implemented in GraphPad Prism 9

$$\frac{I}{I_{\max}}=B_{\max}\times \frac{X^{h}}{{\left(K_{d}\right)}^{h}+X^{h}}$$ (3)

in which *I* represents the mean current at a given concentration of Ca^2+^, *I*_max_ represents the mean current at 40 mM Ca^2+^, *B_max_* represents the saturation value, and *X* represents the concentration of Ca^2+^.

Nonstationary noise analyses to estimate Na^+^ current properties were performed by titrating [Sr^2+^]_free_ from ~2 nM to 6.3 μM. Mean current values, ⟨*I*⟩, and variances, σ^2^, for a given test voltage and concentration of [Sr^2+^]_free_ were calculated by segmenting the pulse (time window: 0.7 to 2.5 s) into 200-ms intervals using Clampfit 10.4. The data points in each noise analysis plot originate from one bilayer. Single-channel current, *i*_Na_, and the number of channels, *n*, were estimated by fitting a plot of σ^2^ versus ⟨*I*⟩ to the following equation using GraphPad Prism 9

$$\sigma^{2}=i_{\mathrm{Na}}\times \langle I\rangle -\frac{{\left\langle I\right\rangle }^{2}}{n}$$ (4)

Open probability, *P*_O_, of the unblocked channel at a given test voltage was subsequently estimated from the following equation, with ⟨*I*⟩ representing the mean current of the unblocked channel

$$P_{o}=\frac{\left\langle I\right\rangle }{i_{\mathrm{Na}}\times n}$$ (5)

Noise analysis was performed using at least three independent experiments (using different bilayers) to estimate *i*_Na_ for *Tc*MCU-EMRE and the mutants.

To estimate single-channel conductance (*g*), *i*_Na_ values deduced from multiple noise analysis experiments were plotted as a function of voltage and fit with a linear regression

$$i_{\mathrm{Na}}=g\times V$$ (6)

AMFE experiments were performed as follows. Starting from symmetric 40 mM Ca^2+^ solutions, the trans solution was perfused with mixtures of Ca^2+^ and Sr^2+^ with a total divalent cation concentration of 40 mM (40 mM Ca^2+^, 34 mM Ca^2+^ and 6 mM Sr^2+^, 28 mM Ca^2+^ and 12 mM Sr^2+^, 20 mM Ca^2+^ and 20 mM Sr^2+^, 12 mM Ca^2+^ and 28 mM Sr^2+^, 6 mM Ca^2+^ and 34 mM Sr^2+^, or 40 mM Sr^2+^). The mean current at −160 mV for each condition was plotted versus the mole fraction of Sr^2+^

$$\text{Mole fraction}=\frac{\left[{Sr}^{2+}\right]}{\left[{Sr}^{2+}]+[{Ca}^{2+}\right]}$$ (7)

The inhibition of Ca^2+^ currents through the channel by La^3+^ was determined by generating currents in 40 mM CaCl_2_ and titrating La(NO_3_)_3_ to the trans chamber (which had been perfused to remove chelators). Mean currents (at −160 mV) recorded in the presence of La^3+^were normalized by dividing by mean currents observed in its absence. The dose response was fit in GraphPad Prism 9 to Eq. 2, with *X* representing the concentration of La^3+^. Nonstationary noise analyses to estimate Ca^2+^ current properties were performed in the same manner as for Na^+^ currents.

## Notes

View/request a protocol for this paper from *Bio-protocol*.

## References

[1] Y. Kirichok, G. Krapivinsky, D. E. Clapham,The mitochondrial calcium uniporter is a highly selective ion channel. Nature 427,360–364 (2004). 14737170 10.1038/nature02246

[2] K. J. Kamer, V. K. Mootha,The molecular era of the mitochondrial calcium uniporter. Nat. Rev. Mol. Cell Biol. 16,545–553 (2015). 26285678 10.1038/nrm4039

[3] X. Pan, J. Liu, T. Nguyen, C. Liu, J. Sun, Y. Teng, M. M. Fergusson, I. I. Rovira, M. Allen, D. A. Springer, A. M. Aponte, M. Gucek, R. S. Balaban, E. Murphy, T. Finkel,The physiological role of mitochondrial calcium revealed by mice lacking the mitochondrial calcium uniporter. Nat. Cell Biol. 15,1464–1472 (2013). 24212091 10.1038/ncb2868 PMC3852190

[4] G. Ashrafi, J. de Juan-Sanz, R. J. Farrell, T. A. Ryan,Molecular tuning of the axonal mitochondrial Ca^2+^ uniporter ensures metabolic flexibility of neurotransmission. Neuron 105,678–687.e5 (2019). 31862210 10.1016/j.neuron.2019.11.020 PMC7035162

[5] P. V. Seegren, T. K. Downs, M. E. Stremska, L. R. Harper, R. Cao, R. J. Olson, C. M. Upchurch, C. A. Doyle, J. Kennedy, E. L. Stipes, N. Leitinger, A. Periasamy, B. N. Desai,Mitochondrial Ca^2+^ signaling is an electrometabolic switch to fuel phagosome killing. Cell Rep. 33,108411 (2020). 33238121 10.1016/j.celrep.2020.108411 PMC7793167

[6] G. W. Dorn II, L. Scorrano,Two close, too close: Sarcoplasmic reticulum-mitochondrial crosstalk and cardiomyocyte fate. Circ. Res. 107,689–699 (2010). 20847324 10.1161/CIRCRESAHA.110.225714 PMC2963937

[7] I. Drago, R. L. Davis,Inhibiting the mitochondrial calcium uniporter during development impairs memory in adult drosophila. Cell Rep. 16,2763–2776 (2016). 27568554 10.1016/j.celrep.2016.08.017 PMC5045571

[8] V. Eisner, G. Csordas, G. Hajnoczky,Interactions between sarco-endoplasmic reticulum and mitochondria in cardiac and skeletal muscle—Pivotal roles in Ca^2+^ and reactive oxygen species signaling. J. Cell Sci. 126,2965–2978 (2013). 23843617 10.1242/jcs.093609 PMC3711195

[9] Y. Sancak, A. L. Markhard, T. Kitami, E. Kovács-Bogdán, K. J. Kamer, N. D. Udeshi, S. A. Carr, D. Chaudhuri, D. E. Clapham, A. A. Li, S. E. Calvo, O. Goldberger, V. K. Mootha,EMRE is an essential component of the mitochondrial calcium uniporter complex. Science 342,1379–1382 (2013). 24231807 10.1126/science.1242993 PMC4091629

[10] D. Chaudhuri, Y. Sancak, V. K. Mootha, D. E. Clapham,MCU encodes the pore conducting mitochondrial calcium currents. eLife 2,e00704 (2013). 23755363 10.7554/eLife.00704 PMC3673318

[11] E. Kovács-Bogdán, Y. Sancak, K. J. Kamer, M. Plovanich, A. Jambhekar, R. J. Huber, M. A. Myre, M. D. Blower, V. K. Mootha,Reconstitution of the mitochondrial calcium uniporter in yeast. Proc. Natl. Acad. Sci. U.S.A. 111,8985–8990 (2014). 24889638 10.1073/pnas.1400514111 PMC4066498

[12] F. Perocchi, V. M. Gohil, H. S. Girgis, X. Robert Bao, J. E. McCombs, A. E. Palmer, V. K. Mootha,MICU1 encodes a mitochondrial EF hand protein required for Ca^2+^ uptake. Nature 467,291–296 (2010). 20693986 10.1038/nature09358 PMC2977980

[13] K. Mallilankaraman, P. Doonan, C. Cárdenas, H. C. Chandramoorthy, M. Müller, R. Miller, N. E. Hoffman, R. K. Gandhirajan, J. Molgó, M. J. Birnbaum, B. S. Rothberg, D. D. Mak, J. K. Foskett, M. Madesh,MICU1 is an essential gatekeeper for MCU-mediated mitochondrial Ca^2+^ uptake that regulates cell survival. Cell 151,630–644 (2012). 23101630 10.1016/j.cell.2012.10.011 PMC3486697

[14] M. Plovanich, R. L. Bogorad, Y. Sancak, K. J. Kamer, L. Strittmatter, A. A. Li, H. S. Girgis, S. Kuchimanchi, J. De Groot, L. Speciner, N. Taneja, J. Oshea, V. Koteliansky, V. K. Mootha,MICU2, a paralog of MICU1, resides within the mitochondrial uniporter complex to regulate calcium handling. PLOS ONE 8,e55785 (2013). 23409044 10.1371/journal.pone.0055785 PMC3567112

[15] K. Mallilankaraman, C. Cárdenas, P. J. Doonan, H. C. Chandramoorthy, K. M. Irrinki, T. Golenár, G. Csordás, P. Madireddi, J. Yang, M. Müller, R. Miller, J. E. Kolesar, J. Molgó, B. Kaufman, G. Hajnóczky, J. Kevin Foskett, M. Madesh,MCUR1 is an essential component of mitochondrial Ca^2+^ uptake that regulates cellular metabolism. Nat. Cell Biol. 14,1336–1343 (2012). 23178883 10.1038/ncb2622 PMC3511605

[16] H. Vais, J. E. Tanis, M. Müller, R. Payne, K. Mallilankaraman, J. K. Foskett,MCUR1, CCDC90A, is a regulator of the mitochondrial calcium uniporter. Cell Metab. 22,533–535 (2015). 26445506 10.1016/j.cmet.2015.09.015 PMC5384258

[17] D. Tomar, Z. Dong, S. Shanmughapriya, D. A. Koch, T. Thomas, N. E. Hoffman, S. A. Timbalia, S. J. Goldman, S. L. Breves, D. P. Corbally, N. Nemani, J. P. Fairweather, A. R. Cutri, X. Zhang, J. Song, F. Jaña, J. Huang, C. Barrero, J. E. Rabinowitz, T. S. Luongo, S. M. Schumacher, M. E. Rockman, A. Dietrich, S. Merali, J. Caplan, P. Stathopulos, R. S. Ahima, J. Y. Cheung, S. R. Houser, W. J. Koch, V. Patel, V. M. Gohil, J. W. Elrod, S. Rajan, M. Madesh,MCUR1 is a scaffold factor for the MCU complex function and promotes mitochondrial bioenergetics. Cell Rep. 15,1673–1685 (2016). 27184846 10.1016/j.celrep.2016.04.050 PMC4880542

[18] A. Raffaello, D. De Stefani, D. Sabbadin, E. Teardo, G. Merli, A. Picard, V. Checchetto, S. Moro, I. Szabò, R. Rizzuto,The mitochondrial calcium uniporter is a multimer that can include a dominant-negative pore-forming subunit. EMBO J. 32,2362–2376 (2013). 23900286 10.1038/emboj.2013.157 PMC3771344

[19] G. Csordás, T. Golenár, E. L. Seifert, K. J. Kamer, Y. Sancak, F. Perocchi, C. Moffat, D. Weaver, S. de la Fuente Perez, R. Bogorad, V. Koteliansky, J. Adijanto, V. K. Mootha, G. Hajnóczky,MICU1 controls both the threshold and cooperative activation of the mitochondrial Ca^2+^ uniporter. Cell Metab. 17,976–987 (2013). 23747253 10.1016/j.cmet.2013.04.020 PMC3722067

[20] K. J. Kamer, Z. Grabarek, V. K. Mootha,High-affinity cooperative Ca^2+^ binding by MICU1–MICU2 serves as an on-off switch for the uniporter. EMBO Rep. 18,1397–1411 (2017). 28615291 10.15252/embr.201643748 PMC5538426

[21] M. Fan, J. Zhang, C. W. Tsai, B. J. Orlando, M. Rodriguez, Y. Xu, M. Liao, M. F. Tsai, L. Feng,Structure and mechanism of the mitochondrial Ca^2+^ uniporter holocomplex. Nature 582,129–133 (2020). 32494073 10.1038/s41586-020-2309-6 PMC7544431

[22] C. Wang, A. Jacewicz, B. D. Delgado, R. Baradaran, S. B. Long,Structures reveal gatekeeping of the mitochondrial Ca^2+^ uniporter by MICU1-MICU2. eLife 9,e59991 (2020). 32667285 10.7554/eLife.59991 PMC7434445

[23] M. Fan, J. Zhang, C.-W. Tsai, B. J. Orlando, M. Rodriguez, Y. Xu, M. Liao, M.-F. Tsai, L. Feng,Structural insights into the Ca^2+^-dependent gating of the human mitochondrial calcium uniporter. eLife 9,e60513 (2020). 32762847 10.7554/eLife.60513 PMC7442490

[24] R. Baradaran, C. Wang, A. F. Siliciano, S. B. Long,Cryo-EM structures of fungal and metazoan mitochondrial calcium uniporters. Nature 559,580–584 (2018). 29995857 10.1038/s41586-018-0331-8 PMC6336196

[25] N. X. Nguyen, J.-P. Armache, C. Lee, Y. Yang, W. Zeng, V. K. Mootha, Y. Cheng, X.-C. Bai, Y. Jiang,Cryo-EM structure of a fungal mitochondrial calcium uniporter. Nature 559,570–574 (2018). 29995855 10.1038/s41586-018-0333-6 PMC6063787

[26] J. Yoo, M. Wu, Y. Yin, M. A. Herzik Jr., G. C. Lander, S.-Y. Lee,Cryo-EM structure of a mitochondrial calcium uniporter. Science 361,506–511 (2018). 29954988 10.1126/science.aar4056 PMC6155975

[27] C. Fan, M. Fan, B. J. Orlando, N. M. Fastman, J. Zhang, Y. Xu, M. G. Chambers, X. Xu, K. Perry, M. Liao, L. Feng,X-ray and cryo-EM structures of the mitochondrial calcium uniporter. Nature 559,575–579 (2018). 29995856 10.1038/s41586-018-0330-9 PMC6368340

[28] Y. Wang, N. X. Nguyen, J. She, W. Zeng, Y. Yang, X.-C. Bai, Y. Jiang,Structural mechanism of EMRE-dependent gating of the human mitochondrial calcium uniporter. Cell 177,1252–1261.e13 (2019). 31080062 10.1016/j.cell.2019.03.050 PMC6597010

[29] C. Wang, R. Baradaran, S. B. Long,Structure and reconstitution of an MCU-EMRE mitochondrial Ca^2+^ uniporter complex. J. Mol. Biol. 432,5632–5648 (2020). 32841658 10.1016/j.jmb.2020.08.013 PMC7577567

[30] J. M. Baughman, F. Perocchi, H. S. Girgis, M. Plovanich, C. A. Belcher-Timme, Y. Sancak, X. R. Bao, L. Strittmatter, O. Goldberger, R. L. Bogorad, V. Koteliansky, V. K. Mootha,Integrative genomics identifies MCU as an essential component of the mitochondrial calcium uniporter. Nature 476,341–345 (2011). 21685886 10.1038/nature10234 PMC3486726

[31] D. De Stefani, A. Raffaello, E. Teardo, I. Szabò, R. Rizzuto,A forty-kilodalton protein of the inner membrane is the mitochondrial calcium uniporter. Nature 476,336–340 (2011). 21685888 10.1038/nature10230 PMC4141877

[32] V. Garg, Y. Y. Kirichok,Patch-clamp analysis of the mitochondrial calcium uniporter. Methods Mol. Biol. 1925,75–86 (2019). 30674018 10.1007/978-1-4939-9018-4_7

[33] C. W. Tsai, M. F. Tsai,Electrical recordings of the mitochondrial calcium uniporter in Xenopus oocytes. J. Gen. Physiol. 150,1035–1043 (2018). 29891485 10.1085/jgp.201812015 PMC6028504

[34] C. Miller, *Ion Channel Reconstitution* (Plenum Press, 1986), pp. xxi, 577.

[35] G. Wu, S. Li, G. Zong, X. Liu, S. Fei, L. Shen, X. Guan, X. Yang, Y. Shen,Single channel recording of a mitochondrial calcium uniporter. Biochem. Biophys. Res. Commun. 496,127–132 (2018). 29307826 10.1016/j.bbrc.2018.01.010

[36] A. Accardi, L. Kolmakova-Partensky, C. Williams, C. Miller,Ionic currents mediated by a prokaryotic homologue of CLC Cl- channels. J. Gen. Physiol. 123,109–119 (2004). 14718478 10.1085/jgp.200308935 PMC2217429

[37] M.-F. Tsai, C. B. Phillips, M. Ranaghan, C.-W. Tsai, Y. Wu, C. Willliams, C. Miller,Dual functions of a small regulatory subunit in the mitochondrial calcium uniporter complex. eLife 5,e15545 (2016). 27099988 10.7554/eLife.15545 PMC4892889

[38] Y. Lee, C. K. Min, T. G. Kim, H. K. Song, Y. Lim, D. Kim, K. Shin, M. Kang, J. Y. Kang, H.-S. Youn, J.-G. Lee, J. Y. An, K. R. Park, J. J. Lim, J. H. Kim, J. H. Kim, Z. Y. Park, Y.-S. Kim, J. Wang, D. H. Kim, S. H. Eom,Structure and function of the N-terminal domain of the human mitochondrial calcium uniporter. EMBO Rep. 16,1318–1333 (2015). 26341627 10.15252/embr.201540436 PMC4662854

[39] K. Oxenoid, Y. Dong, C. Cao, T. Cui, Y. Sancak, A. L. Markhard, Z. Grabarek, L. Kong, Z. Liu, B. Ouyang, Y. Cong, V. K. Mootha, J. J. Chou,Architecture of the mitochondrial calcium uniporter. Nature 533,269–273 (2016). 27135929 10.1038/nature17656 PMC4874835

[40] F. Fieni, S. B. Lee, Y. N. Jan, Y. Kirichok,Activity of the mitochondrial calcium uniporter varies greatly between tissues. Nat. Commun. 3,1317 (2012). 23271651 10.1038/ncomms2325 PMC3818247

[41] V. Garg, J. Suzuki, I. Paranjpe, T. Unsulangi, L. Boyman, L. S. Milescu, W. Jonathan Lederer, Y. Kirichok,The mechanism of MICU-dependent gating of the mitochondrial Ca^2+^uniporter. eLife 10,e69312 (2021). 34463251 10.7554/eLife.69312 PMC8437439

[42] P. Hess, J. B. Lansman, R. W. Tsien,Calcium channel selectivity for divalent and monovalent cations. Voltage and concentration dependence of single channel current in ventricular heart cells. J. Gen. Physiol. 88,293–319 (1986). 2428919 10.1085/jgp.88.3.293 PMC2228831

[43] S. J. Korn, S. R. Ikeda,Permeation selectivity by competition in a delayed rectifier potassium channel. Science 269,410–412 (1995). 7618108 10.1126/science.7618108

[44] P. Hess, R. W. Tsien,Mechanism of ion permeation through calcium channels. Nature 309,453–456 (1984). 6328315 10.1038/309453a0

[45] A. Tinker, A. J. Williams,Divalent cation conduction in the ryanodine receptor channel of sheep cardiac muscle sarcoplasmic reticulum. J. Gen. Physiol. 100,479–493 (1992). 1279095 10.1085/jgp.100.3.479 PMC2229088

[46] I. Bezprozvanny, B. E. Ehrlich,Inositol (1,4,5)-trisphosphate (InsP3)-gated Ca channels from cerebellum: Conduction properties for divalent cations and regulation by intraluminal calcium. J. Gen. Physiol. 104,821–856 (1994). 7876825 10.1085/jgp.104.5.821 PMC2229238

[47] A. V. Yeromin, J. Roos, K. A. Stauderman, M. D. Cahalan,A store-operated calcium channel in Drosophila S2 cells. J. Gen. Physiol. 123,167–182 (2004). 14744989 10.1085/jgp.200308982 PMC2217434

[48] J. K. Foskett, C. White, K.-H. Cheung, D.-O. D. Mak,Inositol trisphosphate receptor Ca^2+^ release channels. Physiol. Rev. 87,593–658 (2007). 17429043 10.1152/physrev.00035.2006 PMC2901638

[49] J. B. Peng, X. Z. Chen, U. V. Berger, P. M. Vassilev, H. Tsukaguchi, E. M. Brown, M. A. Hediger,Molecular cloning and characterization of a channel-like transporter mediating intestinal calcium absorption. J. Biol. Chem. 274,22739–22746 (1999). 10428857 10.1074/jbc.274.32.22739

[50] E. den Dekker, J. G. Hoenderop, B. Nilius, R. J. Bindels,The epithelial calcium channels, TRPV5 & TRPV6: From identification towards regulation. Cell Calcium 33,497–507 (2003). 12765695 10.1016/s0143-4160(03)00065-4

[51] K. J. Kamer, Y. Sancak, Y. Fomina, J. D. Meisel, D. Chaudhuri, Z. Grabarek, V. K. Mootha,MICU1 imparts the mitochondrial uniporter with the ability to discriminate between Ca^2+^ and Mn^2+^. Proc. Natl. Acad. Sci. U.S.A. 115,E7960–E7969 (2018). 30082385 10.1073/pnas.1807811115 PMC6112746

[52] J. Wettmarshausen, V. Goh, K.-T. Huang, D. M. Arduino, U. Tripathi, A. Leimpek, Y. Cheng, A. A. Pittis, T. Gabaldón, D. Mokranjac, G. Hajnóczky, F. Perocchi,MICU1 confers protection from MCU-dependent manganese toxicity. Cell Rep. 25,1425–1435.e7 (2018). 30403999 10.1016/j.celrep.2018.10.037

[53] C. B. Phillips, C. W. Tsai, M. F. Tsai,The conserved aspartate ring of MCU mediates MICU1 binding and regulation in the mitochondrial calcium uniporter complex. eLife 8,e41112 (2019). 30638448 10.7554/eLife.41112 PMC6347451

[54] R. J. French, J. B. Wells,Sodium ions as blocking agents and charge carriers in the potassium channel of the squid giant axon. J. Gen. Physiol. 70,707–724 (1977). 591920 10.1085/jgp.70.6.707 PMC2228512

[55] B. Hille, *Ionic Channels of Excitable Membranes* (Sinauer Associates, ed. 2, 1992), pp. xiii, p. 607.

[56] C. M. Nimigean, C. Miller,Na^+^ block and permeation in a K^+^ channel of known structure. J. Gen. Physiol. 120,323–335 (2002). 12198089 10.1085/jgp.20028614 PMC2229518

[57] M. Prakriya, R. S. Lewis,Regulation of CRAC channel activity by recruitment of silent channels to a high open-probability gating mode. J. Gen. Physiol. 128,373–386 (2006). 16940559 10.1085/jgp.200609588 PMC2151560

[58] G. Eisenman, R. Horn,Ionic selectivity revisited: The role of kinetic and equilibrium processes in ion permeation through channels. J. Membr. Biol. 76,197–225 (1983). 6100862 10.1007/BF01870364

[59] W. Zhuo, H. Zhou, R. Guo, J. Yi, L. Yu, Y. Sui, L. Zhang, W. Zeng, P. Wang, M. Yang, Structure of intact human MCU supercomplex with the auxiliary MICU subunits. bioRxiv 025205 [Preprint]. 6 April 2020. 10.1101/2020.04.04.025205. PMC7895871 32862359

[60] F. J. Sigworth,The variance of sodium current fluctuations at the node of Ranvier. J. Physiol. 307,97–129 (1980). 6259340 10.1113/jphysiol.1980.sp013426 PMC1283036

[61] X. Tao, R. K. Hite, R. MacKinnon,Cryo-EM structure of the open high-conductance Ca^2+^-activated K^+^ channel. Nature 541,46–51 (2017). 27974795 10.1038/nature20608 PMC5500982

[62] B. Hille, W. Schwarz,Potassium channels as multi-ion single-file pores. J. Gen. Physiol. 72,409–442 (1978). 722275 10.1085/jgp.72.4.409 PMC2228548

[63] J. Bormann, O. P. Hamill, B. Sakmann,Mechanism of anion permeation through channels gated by glycine and gamma-aminobutyric acid in mouse cultured spinal neurones. J. Physiol. 385,243–286 (1987). 2443667 10.1113/jphysiol.1987.sp016493 PMC1192346

[64] C. Miller,Ionic hopping defended. J. Gen. Physiol. 113,783–787 (1999). 10352029 10.1085/jgp.113.6.783 PMC2225609

[65] E. W. McCleskey,Calcium channel permeation: A field in flux. J. Gen. Physiol. 113,765–772 (1999). 10352027 10.1085/jgp.113.6.765 PMC2225606

[66] W. A. Sather, E. W. McCleskey,Permeation and selectivity in calcium channels. Annu. Rev. Physiol. 65,133–159 (2003). 12471162 10.1146/annurev.physiol.65.092101.142345

[67] D. A. Doyle, J. Morais Cabral, R. A. Pfuetzner, A. Kuo, J. M. Gulbis, S. L. Cohen, B. T. Chait, R. MacKinnon,The structure of the potassium channel: Molecular basis of K^+^ conduction and selectivity. Science 280,69–77 (1998). 9525859 10.1126/science.280.5360.69

[68] J. H. Morais-Cabral, Y. Zhou, R. Mackinnon,Energetic optimization of ion conduction rate by the K^+^ selectivity filter. Nature 414,37–42 (2001). 11689935 10.1038/35102000

[69] S. B. Long, E. B. Campbell, R. MacKinnon,Crystal structure of a mammalian voltage-dependent shaker family K^+^ channel. Science 309,897–903 (2005). 16002581 10.1126/science.1116269

[70] S. B. Long, X. Tao, E. B. Campbell, R. MacKinnon,Atomic structure of a voltage-dependent K^+^ channel in a lipid membrane-like environment. Nature 450,376–382 (2007). 18004376 10.1038/nature06265

[71] S. W. Lockless, M. Zhou, R. MacKinnon,Structural and thermodynamic properties of selective ion binding in a K^+^ channel. PLoS Biol. 5,e121 (2007). 17472437 10.1371/journal.pbio.0050121 PMC1858713

[72] A. Lepple-Wienhues, M. D. Cahalan,Conductance and permeation of monovalent cations through depletion-activated Ca^2+^ channels (ICRAC) in Jurkat T cells. Biophys. J. 71,787–794 (1996). 8842217 10.1016/S0006-3495(96)79278-0 PMC1233535

[73] A. Zweifach, R. S. Lewis,Mitogen-regulated Ca^2+^ current of T lymphocytes is activated by depletion of intracellular Ca^2+^ stores. Proc. Natl. Acad. Sci. U.S.A. 90,6295–6299 (1993). 8392195 10.1073/pnas.90.13.6295 PMC46915

[74] M. Prakriya, R. S. Lewis,Separation and characterization of currents through store-operated CRAC channels and Mg^2+^-inhibited cation (MIC) channels. J. Gen. Physiol. 119,487–507 (2002). 11981025 10.1085/jgp.20028551 PMC2233817

[75] H. A. Lester, D. A. Dougherty,New views of multi-ion channels. J. Gen. Physiol. 111,181–183 (1998). 9450937 10.1085/jgp.111.2.181 PMC2222761

[76] S. Sakipov, A. I. Sobolevsky, M. G. Kurnikova,Ion permeation mechanism in epithelial calcium channel TRVP6. Sci. Rep. 8,5715 (2018). 29632318 10.1038/s41598-018-23972-5 PMC5890290

[77] D. D. Friel, R. W. Tsien,Voltage-gated calcium channels: Direct observation of the anomalous mole fraction effect at the single-channel level. Proc. Natl. Acad. Sci. U.S.A. 86,5207–5211 (1989). 2544893 10.1073/pnas.86.13.5207 PMC297587

[78] E. Gouaux, R. Mackinnon,Principles of selective ion transport in channels and pumps. Science 310,1461–1465 (2005). 16322449 10.1126/science.1113666

[79] Y. Zhou, J. H. Morais-Cabral, A. Kaufman, R. Mackinnon,Chemistry of ion coordination and hydration revealed by a K^+^ channel-Fab complex at 2.0 A resolution. Nature 414,43–48 (2001). 11689936 10.1038/35102009

[80] F. H. Yu, V. Yarov-Yarovoy, G. A. Gutman, W. A. Catterall,Overview of molecular relationships in the voltage-gated ion channel superfamily. Pharmacol. Rev. 57,387–395 (2005). 16382097 10.1124/pr.57.4.13

[81] J. Wu, Z. Yan, Z. Li, X. Qian, S. Lu, M. Dong, Q. Zhou, N. Yan,Structure of the voltage-gated calcium channel Ca(v)1 at 3.6 Å resolution. Nature 537,191–196 (2016). 27580036 10.1038/nature19321

[82] X. Pan, Z. Li, Q. Zhou, H. Shen, K. Wu, X. Huang, J. Chen, J. Zhang, X. Zhu, J. Lei, W. Xiong, H. Gong, B. Xiao, N. Yan,Structure of the human voltage-gated sodium channel Nav1.4 in complex with β1. Science 362,eaau2486 (2018). 30190309 10.1126/science.aau2486

[83] Y. Zhou, R. MacKinnon,The occupancy of ions in the K^+^ selectivity filter: Charge balance and coupling of ion binding to a protein conformational change underlie high conduction rates. J. Mol. Biol. 333,965–975 (2003). 14583193 10.1016/j.jmb.2003.09.022

[84] B. Li, R. A. Rietmeijer, S. G. Brohawn,Structural basis for pH gating of the two-pore domain K^+^ channel TASK2. Nature 586,457–462 (2020). 32999458 10.1038/s41586-020-2770-2 PMC8628578

[85] X.-F. Tan, C. Bae, R. Stix, A. I. Fernández-Mariño, K. Huffer, T.-H. Chang, J. Jiang, J. D. Faraldo-Gómez, K. J. Swartz,Structure of the Shaker Kv channel and mechanism of slow C-type inactivation. Sci. Adv. 8,eabm7814 (2022). 35302848 10.1126/sciadv.abm7814 PMC8932672

[86] J. Park, Y. Lee, T. Park, J. Y. Kang, S. A. Mun, M. Jin, J. Yang, S. H. Eom,Structure of the MICU1-MICU2 heterodimer provides insights into the gatekeeping threshold shift. IUCrJ 7,355–365 (2020). 10.1107/S2052252520001840 PMC7055370 32148862

[87] R. MacKinnon, R. Latorre, C. Miller,Role of surface electrostatics in the operation of a high-conductance Ca^2+^-activated K^+^ channel. Biochemistry 28,8092–8099 (1989). 2605175 10.1021/bi00446a020

[88] M. Paillard, G. Csordás, K.-T. Huang, P. Várnai, S. K. Joseph, G. Hajnóczky,MICU1 interacts with the D-ring of the MCU pore to control its Ca^2+^ flux and sensitivity to Ru360. Mol. Cell 72,778–785.e3 (2018). 30454562 10.1016/j.molcel.2018.09.008 PMC6251499

[89] A. L. Hodgkin, R. D. Keynes,The potassium permeability of a giant nerve fibre. J. Physiol. 128,61–88 (1955). 10.1113/jphysiol.1955.sp005291 PMC1365755 14368575

[90] G. Eisenman, J. P. Sandblom, J. L. Walker Jr.,Membrane structure and ion permeation. Study of ion exchange membrane structure and function is relevant to analysis of biological ion permeation. Science 155,965–974 (1967). 5334938 10.1126/science.155.3765.965

[91] T. X. Dang, E. W. McCleskey,Ion channel selectivity through stepwise changes in binding affinity. J. Gen. Physiol. 111,185–193 (1998). 9450938 10.1085/jgp.111.2.185 PMC2222772

[92] D. E. Clapham,TRP channels as cellular sensors. Nature 426,517–524 (2003). 14654832 10.1038/nature02196

[93] A. V. Yeromin, S. L. Zhang, W. Jiang, Y. Yu, O. Safrina, M. D. Cahalan,Molecular identification of the CRAC channel by altered ion selectivity in a mutant of Orai. Nature 443,226–229 (2006). 16921385 10.1038/nature05108 PMC2756048

[94] X. Hou, L. Pedi, M. M. Diver, S. B. Long,Crystal structure of the calcium release-activated calcium channel Orai. Science 338,1308–1313 (2012). 23180775 10.1126/science.1228757 PMC3695727

[95] A. Jairaman, M. Prakriya,Molecular pharmacology of store-operated CRAC channels. Channels (Austin) 7,402–414 (2013). 23807116 10.4161/chan.25292 PMC3913763

[96] K. Saotome, A. K. Singh, M. V. Yelshanskaya, A. I. Sobolevsky,Crystal structure of the epithelial calcium channel TRPV6. Nature 534,506–511 (2016). 27296226 10.1038/nature17975 PMC4919205

[97] T. Kawate, E. Gouaux,Fluorescence-detection size-exclusion chromatography for precrystallization screening of integral membrane proteins. Structure 14,673–681 (2006). 16615909 10.1016/j.str.2006.01.013

[98] A. Kirchhofer, J. Helma, K. Schmidthals, C. Frauer, S. Cui, A. Karcher, M. Pellis, S. Muyldermans, C. S. Casas-Delucchi, M. C. Cardoso, H. Leonhardt, K.-P. Hopfner, U. Rothbauer,Modulation of protein properties in living cells using nanobodies. Nat. Struct. Mol. Biol. 17,133–138 (2010). 20010839 10.1038/nsmb.1727

[99] W. J. Degrip, J. Vanoostrum, P. H. Bovee-Geurts,Selective detergent-extraction from mixed detergent/lipid/protein micelles, using cyclodextrin inclusion compounds: A novel generic approach for the preparation of proteoliposomes. Biochem. J. 330 (Pt 2),667–674 (1998). 9480873 10.1042/bj3300667 PMC1219188

[100] G. Vaisey, S. B. Long,An allosteric mechanism of inactivation in the calcium-dependent chloride channel BEST1. J. Gen. Physiol. 150,1484–1497 (2018). 30237227 10.1085/jgp.201812190 PMC6219684

[101] Y. Marcus,Thermodynamics of solvation of ions. Part 5.—Gibbs free energy of hydration at 298.15 K. J. Chem. Soc. Faraday Trans. 87,2995–2999 (1991).

