# Supplementary material for: Mechanisms of ion selectivity and throughput in the mitochondrial calcium uniporter
Source: Sci Adv. 2022 Dec 16;8(50):eade1516. doi: 10.1126/sciadv.ade1516 (PMC9757755; doi:10.1126/sciadv.ade1516)
Supplement: Supplementary file 1 — Figs. S1 to S6 Tables S1 to S9 [file sciadv.ade1516_sm.pdf]

Science Advances
AAAS

Supplementary Materials for

Mechanisms of ion selectivity and throughput in the mitochondrial calcium uniporter

Bryce D. Delgado and Stephen B. Long

Corresponding author: Stephen B. Long, longs@mskcc.org

Sci. Adv. **8**, eade1516 (2022)
DOI: 10.1126/sciadv.ade1516

This PDF file includes:

Figs. S1 to S6
Tables S1 to S9

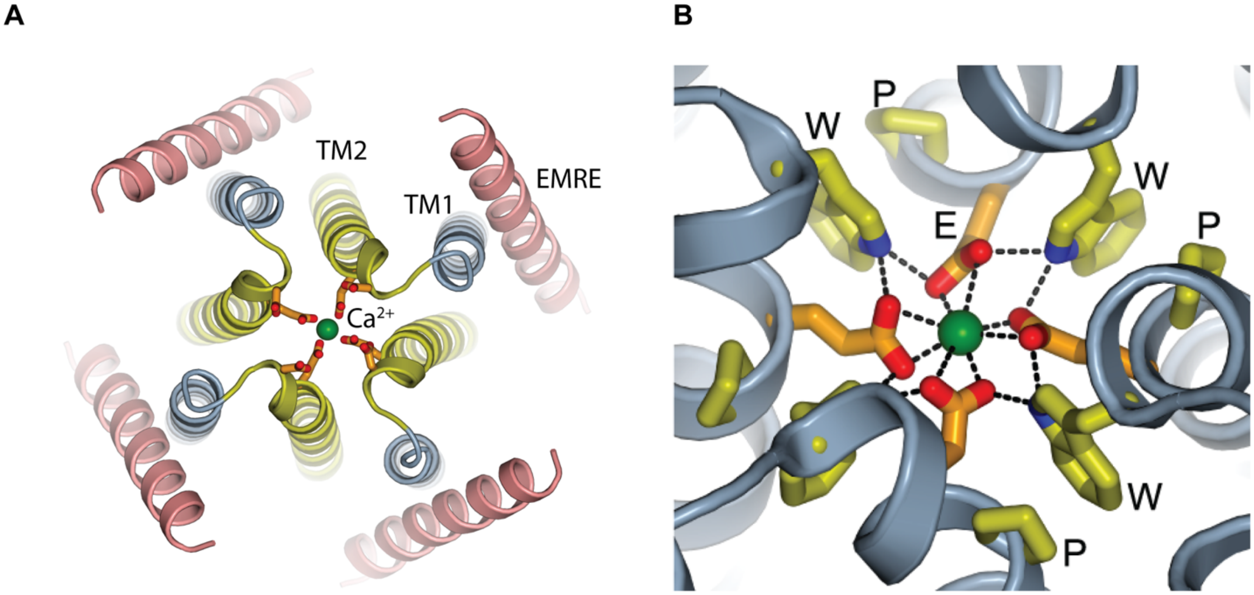

Fig. S1. Subunit assembly and stabilization of the E-locus. (A) View of *Tc*MCU-EMRE (PDB: 6X4S, cartoon representation) from the IMS. The D- and E-locus residues are drawn as sticks; Ca^{2+} is depicted as a green sphere. Helices are labeled. **(B)** Atomic model of the E-locus region demonstrates how the neighboring tryptophan ('W') and proline ('P') residues stabilize the glutamate side chains ('E'). Hydrogen bonds and interactions with the Ca^{2+} ion are shown as dotted lines.

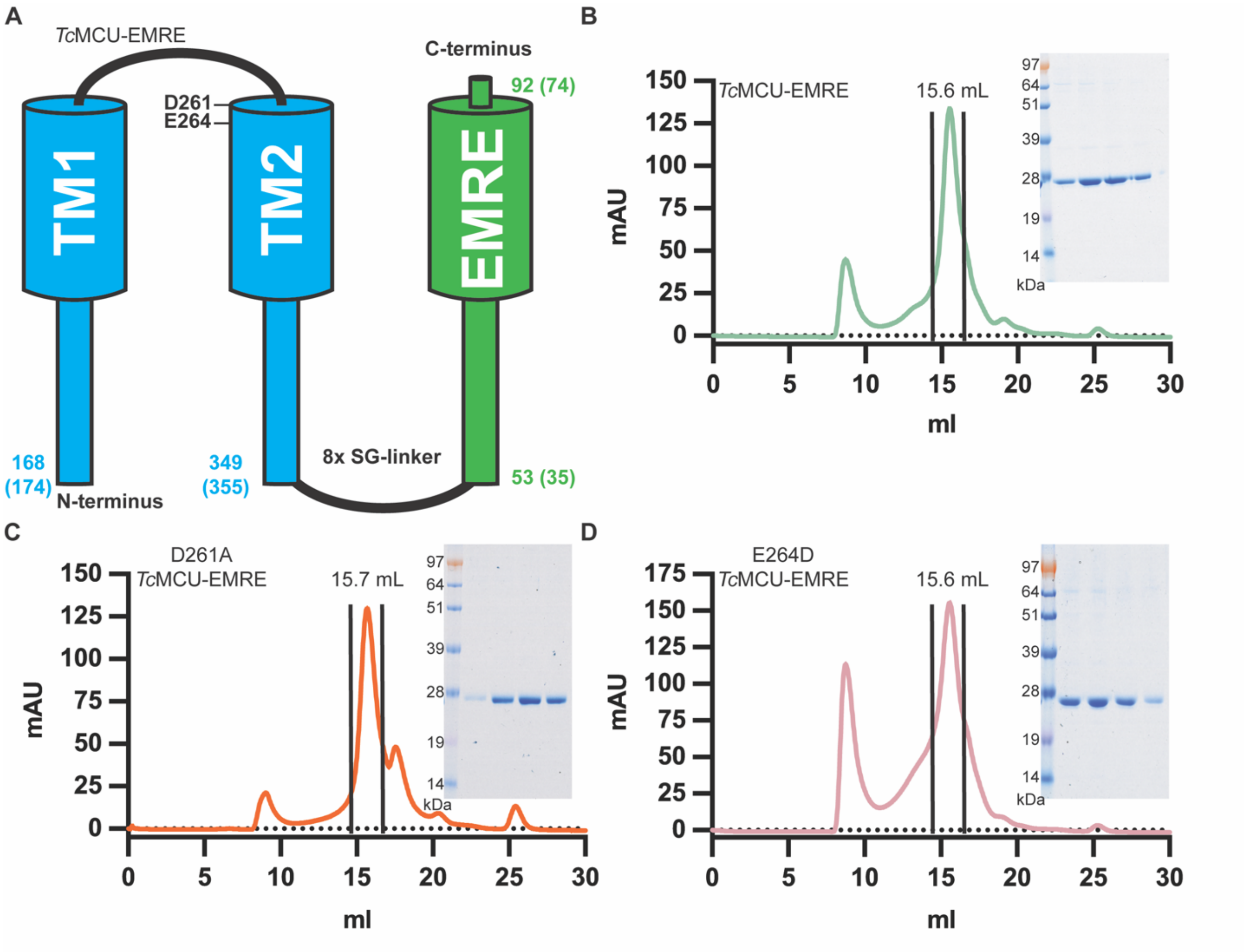

Fig. S2. *Tc*MCU-EMRE construct and purification. (A) Schematic of the *Tc*MCU-EMRE construct used for electrical recordings. Human numbering is shown, with *Tribolium castaneum* numbering indicated in paratheses. **(B-D)** Size exclusion chromatography (SEC) and SDS-PAGE (Coomassie stained) analyses of the protein samples. The SEC fractions used for reconstitution are denoted by vertical lines and correspond to samples analyzed by SDS-PAGE. Retention volumes are indicated above the peaks.

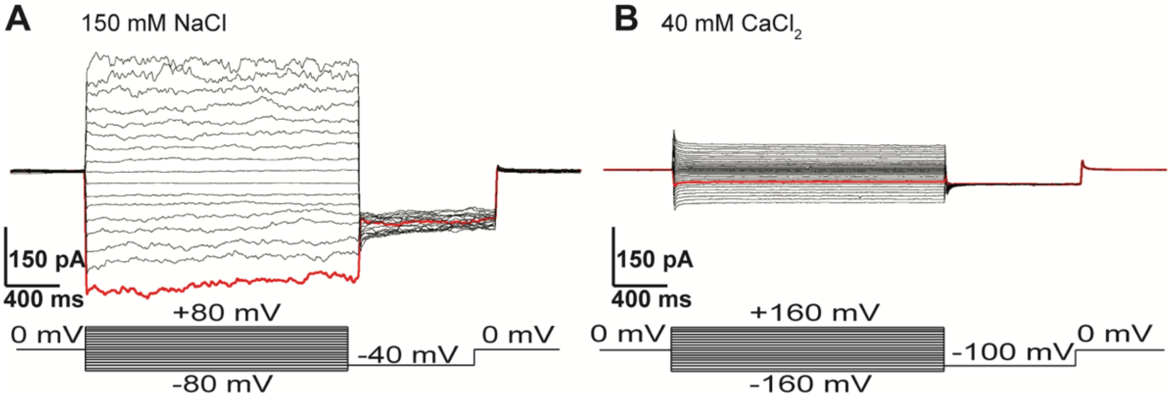

Fig. S3. Macroscopic recordings of Na^+ and Ca^{2+} currents through *Tc*MCU-EMRE channels from the same membrane. (A & B) Electrical recordings for Na^+ currents (A, 150 mM NaCl) and Ca^{2+} **(**B, 40 mM $CaCl_2$) from the same bilayer demonstrate differences in current magnitude. The voltage protocols are indicated below the current traces. The red traces highlight that the Na^+ current evoked by a voltage step to -80 mV is approximately ten times greater than the Ca^{2+} current at -80 mV.

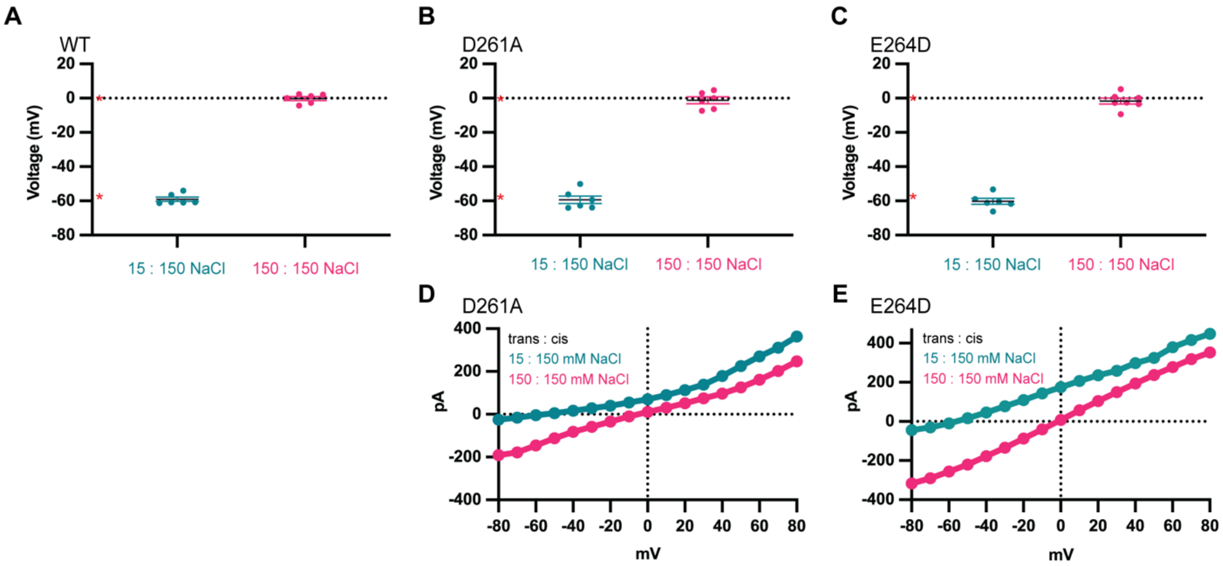

Fig. S4. Cation-versus-anion selectivity of *Tc*MCU-EMRE and mutants. (A-E) Reversal potentials demonstrate selectivity for Na^+ over Cl^- in all three constructs. Reversal potentials (plotted in A-C) were determined from I-V plots as those shown in (D & E and in Fig. 1C) for symmetric (150 mM) and asymmetric (15 mM trans : 150 mM cis) NaCl concentrations under divalent-free conditions. Nernst potentials for the symmetric and asymmetric conditions (0 and -58 mV, respectively) are denoted on the *Y*-axes in (A-C) by red asterisks. Each point in (A-C) represents a separate experiment from a different membrane, with error bars representing the SEM.

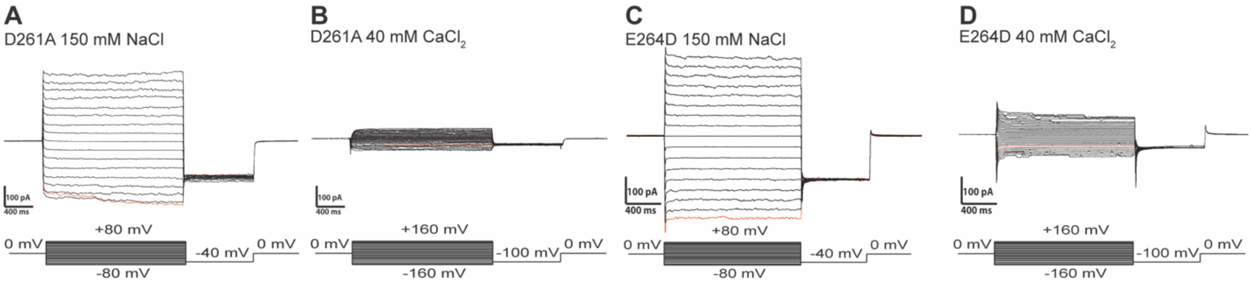

Fig. S5. Macroscopic recordings of Na^+ and Ca^{2+} currents of the mutant proteins. (A - D) Electrical recordings for Na^+ currents (A & C, 150 mM NaCl) and Ca^{2+} (B & D, 40 mM $CaCl_2$) from the same bilayer demonstrate differences in current magnitude for the indicated mutant. The voltage protocols are given below the current traces. The red traces highlight that the Na^+ current evoked by a voltage step to -80 mV is greater than the Ca^{2+} current at -80 mV.

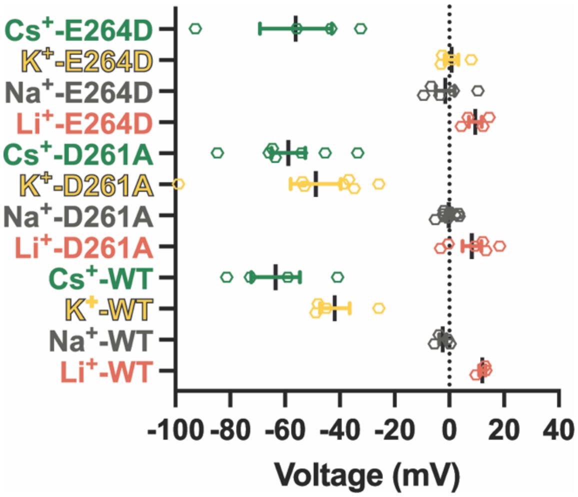

Fig. S6. Reversal potentials determined from symmetric and bi-ionic monovalent cation experiments. Reversal potentials from *Tc*MCU-EMRE (WT) and mutant channels were determined from experiments represented in Figure 5A-C. Test cations on the trans side (at 150 mM, as Cl^- salts) are indicated. 150 mM NaCl was used on the cis side in each experiment. Each point represents a separate experiment using a different bilayer. Error bars represent SEM.

Panel	Trans Chamber	Cis Chamber
C, Grey line	15 mM NaCl, 1 mM EGTA-NMDG, 1 mM EDTA-NMDG, 20 mM HEPES-NMDG, pH 7.6	150 mM NaCl, 1 mM EGTA-NMDG, 1 mM EDTA-NMDG, 20 mM HEPES-NMDG, pH 7.6
C, Green line	150 mM NaCl, 1 mM EGTA-NMDG, 1 mM EDTA-NMDG, 20 mM HEPES-NMDG, pH 7.6	150 mM NaCl, 1 mM EGTA-NMDG, 1 mM EDTA-NMDG, 20 mM HEPES-NMDG, pH 7.6
D	150 mM NaCl, 1 mM EGTA-NMDG, 1 mM EDTA-NMDG, 20 mM HEPES-NMDG, pH 7.6	150 mM NaCl, 1 mM EGTA-NMDG, 1 mM EDTA-NMDG, 20 mM HEPES-NMDG, pH 7.6
E	150 mM NaCl, 1 mM EGTA-NMDG, 1 mM EDTA-NMDG, 20 mM HEPES-NMDG, 10 nM $[CaCl_2]_{free}$, pH 7.6	150 mM NaCl, 1 mM EGTA-NMDG, 1 mM EDTA-NMDG, 20 mM HEPES-NMDG, 10 nM $[CaCl_2]_{free}$, pH 7.6
F, Blue line	150 mM KCl, 1 mM EGTA-NMDG, 1 mM EDTA-NMDG, 20 mM HEPES-NMDG, pH 7.6	150 mM NaCl, 1 mM EGTA-NMDG, 1 mM EDTA-NMDG, 20 mM HEPES-NMDG, pH 7.6
F, Orange line	150 mM NaCl, 1 mM EGTA-NMDG, 1 mM EDTA-NMDG, 20 mM HEPES-NMDG, pH 7.6	150 mM NaCl, 1 mM EGTA-NMDG, 1 mM EDTA-NMDG, 20 mM HEPES-NMDG, pH 7.6

Table S1. Buffer compositions for experiments shown in Figure 1 (Monovalent currents of *Tc*MCU-EMRE).

Panel	Trans Chamber	Cis Chamber
A	40 mM $CaCl_2$, 1 mM EGTA-NMDG, 1 mM EDTA-NMDG, 20 mM HEPES-NMDG, pH 7.6	40 mM $CaCl_2$, 150 mM NaCl, 1 mM EGTA-NMDG, 1 mM EDTA-NMDG, 20 mM HEPES-NMDG, pH 7.6
B, Red curve	150 mM NaCl, 1 mM EGTA-NMDG, 1 mM EDTA-NMDG, 20 mM HEPES-NMDG, pH 7.6	40 mM $CaCl_2$, 150 mM NaCl, 1 mM EGTA-NMDG, 1 mM EDTA-NMDG, 20 mM HEPES-NMDG, pH 7.6
B, Blue curve	150 mM KCl, 1 mM EGTA-NMDG, 1 mM EDTA-NMDG, 20 mM HEPES-NMDG, pH 7.6	40 mM $CaCl_2$, 150 mM NaCl, 1 mM EGTA-NMDG, 1 mM EDTA-NMDG, 20 mM HEPES-NMDG, pH 7.6
C	40 mM XCl_2, 1 mM EGTA-NMDG, 1 mM EDTA-NMDG, 20 mM HEPES-NMDG, pH 7.6<br><br>X: (Ca^{2+}, Sr^{2+}, Mn^{2+}, Ba^{2+}, or Mg^{2+})	40 mM $CaCl_2$, 150 mM NaCl, 1 mM EGTA-NMDG, 1 mM EDTA-NMDG, 20 mM HEPES-NMDG, pH 7.6
E	40 mM $CaCl_2$, 1 mM EGTA-NMDG, 1 mM EDTA-NMDG, 20 mM HEPES-NMDG, pH 7.6	40 mM $CaCl_2$, 150 mM NaCl, 1 mM EGTA-NMDG, 1 mM EDTA-NMDG, 20 mM HEPES-NMDG, 1 μM RuRed pH 7.6
F	40 mM $CaCl_2$, 1 mM EGTA-NMDG, 1 mM EDTA-NMDG, 20 mM HEPES-NMDG, 1 μM RuRed, pH 7.6	40 mM $CaCl_2$, 150 mM NaCl, 1 mM EGTA-NMDG, 1 mM EDTA-NMDG, 20 mM HEPES-NMDG, 1 μM RuRed, pH 7.6
G, Blue curve	40 mM $CaCl_2$, 1 mM EGTA-NMDG, 1 mM EDTA-NMDG, 20 mM HEPES-NMDG, pH 7.6	40 mM $CaCl_2$, 150 mM NaCl, 1 mM EGTA-NMDG, 1 mM EDTA-NMDG, 20 mM HEPES-NMDG, pH 7.6
G, Black curve	40 mM $CaCl_2$, 1 mM EGTA-NMDG, 1 mM EDTA-NMDG, 20 mM HEPES-NMDG, pH 7.6	40 mM $CaCl_2$, 150 mM NaCl, 1 mM EGTA-NMDG, 1 mM EDTA-NMDG, 20 mM HEPES-NMDG, 1 μM RuRed pH 7.6

Table S2. Buffer compositions for Figure 2 (Ca^{2+} selectivity and RuRed inhibition of *Tc*MCU-EMRE).

Panel	Trans Chamber	Cis Chamber
A	150 mM NaCl, 1 mM EGTA-NMDG, 1 mM EDTA-NMDG, 20 mM HEPES-NMDG, pH 7.6	150 mM NaCl, 1 mM EGTA-NMDG, 1 mM EDTA-NMDG, 20 mM HEPES-NMDG, pH 7.6
B	150 mM NaCl, 1 mM EGTA-NMDG, 1 mM EDTA-NMDG, 20 mM HEPES-NMDG, 10 nM $[CaCl_2]_{free}$, pH 7.6	150 mM NaCl, 1 mM EGTA-NMDG, 1 mM EDTA-NMDG, 20 mM HEPES-NMDG, 10 nM $[CaCl_2]_{free}$, pH 7.6
C	150 mM NaCl, 1 mM EGTA-NMDG, 1 mM EDTA-NMDG, 20 mM HEPES-NMDG, pH 7.6	150 mM NaCl, 1 mM EGTA-NMDG, 1 mM EDTA-NMDG, 20 mM HEPES-NMDG, pH 7.6
D	150 mM NaCl, 1 mM EGTA-NMDG, 1 mM EDTA-NMDG, 20 mM HEPES-NMDG, 1 µM $[CaCl_2]_{free}$, pH 7.6	150 mM NaCl, 1 mM EGTA-NMDG, 1 mM EDTA-NMDG, 20 mM HEPES-NMDG, 1 µM $[CaCl_2]_{free}$, pH 7.6
E, Ca^{2+} titration	150 mM NaCl, 1 mM EGTA-NMDG, 1 mM EDTA-NMDG, 20 mM HEPES-NMDG, 0-100 µM $[CaCl_2]_{free}$, pH 7.6	150 mM NaCl, 1 mM EGTA-NMDG, 1 mM EDTA-NMDG, 20 mM HEPES-NMDG, 0-100 µM $[CaCl_2]_{free}$, pH 7.6
F, Sr^{2+} titration	150 mM NaCl, 1 mM EGTA-NMDG, 1 mM EDTA-NMDG, 20 mM HEPES-NMDG, 0-6.3 µM $[SrCl_2]_{free}$, pH 7.6	150 mM NaCl, 1 mM EGTA-NMDG, 1 mM EDTA-NMDG, 20 mM HEPES-NMDG, 0-6.3 µM $[SrCl_2]_{free}$, pH 7.6
G & H	150 mM NaCl, 1 mM EGTA-NMDG, 1 mM EDTA-NMDG, 20 mM HEPES-NMDG, 10 nM $[SrCl_2]_{free}$, pH 7.6	150 mM NaCl, 1 mM EGTA-NMDG, 1 mM EDTA-NMDG, 20 mM HEPES-NMDG, 10 nM $[SrCl_2]_{free}$, pH 7.6
I	150 mM NaCl, 1 mM EGTA-NMDG, 1 mM EDTA-NMDG, 20 mM HEPES-NMDG, 1 µM $[SrCl_2]_{free}$, pH 7.6	150 mM NaCl, 1 mM EGTA-NMDG, 1 mM EDTA-NMDG, 20 mM HEPES-NMDG, 1 µM $[SrCl_2]_{free}$, pH 7.6

Table S3. Buffer compositions for Figure 3 (E-locus is a high-affinity binding site for Ca^{2+}). For $[Ca^{2+}]_{free}$ and $[Sr^{2+}]_{free}$, MaxChelator was used to determine the amount of $CaCl_2$ or $SrCl_2$ added to reach the desired value (Methods).

Panel	Trans Chamber	Cis Chamber
A-E, Ca^{2+} condition	40 mM $CaCl_2$, 1 mM EGTA-NMDG, 1 mM EDTA-NMDG, 20 mM HEPES-NMDG, pH 7.6	40 mM $CaCl_2$, 150 mM NaCl, 1 mM EGTA-NMDG, 1 mM EDTA-NMDG, 20 mM HEPES-NMDG, pH 7.6
A-E, Sr^{2+} condition	40 mM $SrCl_2$, 1 mM EGTA-NMDG, 1 mM EDTA-NMDG, 20 mM HEPES-NMDG, pH 7.6	40 mM $CaCl_2$, 150 mM NaCl, 1 mM EGTA-NMDG, 1 mM EDTA-NMDG, 20 mM HEPES-NMDG, pH 7.6
A-E, Ba^{2+} condition	40 mM $BaCl_2$, 1 mM EGTA-NMDG, 1 mM EDTA-NMDG, 20 mM HEPES-NMDG, pH 7.6	40 mM $CaCl_2$, 150 mM NaCl, 1 mM EGTA-NMDG, 1 mM EDTA-NMDG, 20 mM HEPES-NMDG, pH 7.6
A-E, Mn^{2+} condition	40 mM $MnCl_2$, 1 mM EGTA-NMDG, 1 mM EDTA-NMDG, 20 mM HEPES-NMDG, pH 7.6	40 mM $CaCl_2$, 150 mM NaCl, 1 mM EGTA-NMDG, 1 mM EDTA-NMDG, 20 mM HEPES-NMDG, pH 7.6
A-E, Mg^{2+} condition	40 mM $MgCl_2$, 1 mM EGTA-NMDG, 1 mM EDTA-NMDG, 20 mM HEPES-NMDG, pH 7.6	40 mM $CaCl_2$, 150 mM NaCl, 1 mM EGTA-NMDG, 1 mM EDTA-NMDG, 20 mM HEPES-NMDG, pH 7.6

Table S4. Buffer compositions for Figure 4 (Divalent selectivity of mutant channels).

Panel	Trans Chamber	Cis Chamber
A-E, Li^+ condition	150 mM LiCl, 1 mM EGTA-NMDG, 1 mM EDTA-NMDG, 20 mM HEPES-NMDG, pH 7.6	150 mM NaCl, 1 mM EGTA-NMDG, 1 mM EDTA-NMDG, 20 mM HEPES-NMDG, pH 7.6
A-E, Na^+ condition	150 mM NaCl, 1 mM EGTA-NMDG, 1 mM EDTA-NMDG, 20 mM HEPES-NMDG, pH 7.6	150 mM NaCl, 1 mM EGTA-NMDG, 1 mM EDTA-NMDG, 20 mM HEPES-NMDG, pH 7.6
A-E, K^+ condition	150 mM KCl, 1 mM EGTA-NMDG, 1 mM EDTA-NMDG, 20 mM HEPES-NMDG, pH 7.6	150 mM NaCl, 1 mM EGTA-NMDG, 1 mM EDTA-NMDG, 20 mM HEPES-NMDG, pH 7.6
A-E, Cs^+ condition	150 mM CsCl, 1 mM EGTA-NMDG, 1 mM EDTA-NMDG, 20 mM HEPES-NMDG, pH 7.6	150 mM NaCl, 1 mM EGTA-NMDG, 1 mM EDTA-NMDG, 20 mM HEPES-NMDG, pH 7.6

Table S5. Buffer compositions for Figure 5 (Monovalent selectivity of *Tc*MCU-EMRE and mutants).

Panel	Trans Chamber	Cis Chamber
A, Black trace	150 mM NaCl, 1 mM EGTA-NMDG, 1 mM EDTA-NMDG, 20 mM HEPES-NMDG, pH 7.6	150 mM NaCl, 1 mM EGTA-NMDG, 1 mM EDTA-NMDG, 20 mM HEPES-NMDG, pH 7.6
A, Navy trace	150 mM NaCl, 1 mM EGTA-NMDG, 1 mM EDTA-NMDG, 20 mM HEPES-NMDG, 8.4 nM $[SrCl_2]_{free}$, pH 7.6	150 mM NaCl, 1 mM EGTA-NMDG, 1 mM EDTA-NMDG, 20 mM HEPES-NMDG, 8.4 nM $[SrCl_2]_{free}$, pH 7.6
A, Red trace	150 mM NaCl, 1 mM EGTA-NMDG, 1 mM EDTA-NMDG, 20 mM HEPES-NMDG, 3.8 µM $[SrCl_2]_{free}$, pH 7.6	150 mM NaCl, 1 mM EGTA-NMDG, 1 mM EDTA-NMDG, 20 mM HEPES-NMDG, 3.8 µM $[SrCl_2]_{free}$, pH 7.6
B, Black trace	150 mM NaCl, 1 mM EGTA-NMDG, 1 mM EDTA-NMDG, 20 mM HEPES-NMDG, pH 7.6	150 mM NaCl, 1 mM EGTA-NMDG, 1 mM EDTA-NMDG, 20 mM HEPES-NMDG, pH 7.6
B, Navy trace	150 mM NaCl, 1 mM EGTA-NMDG, 1 mM EDTA-NMDG, 20 mM HEPES-NMDG, 8.4 nM $[SrCl_2]_{free}$, pH 7.6	150 mM NaCl, 1 mM EGTA-NMDG, 1 mM EDTA-NMDG, 20 mM HEPES-NMDG, 8.4 nM $[SrCl_2]_{free}$, pH 7.6
B, Red trace	150 mM NaCl, 1 mM EGTA-NMDG, 1 mM EDTA-NMDG, 20 mM HEPES-NMDG, 3.8 µM $[SrCl_2]_{free}$, pH 7.6	150 mM NaCl, 1 mM EGTA-NMDG, 1 mM EDTA-NMDG, 20 mM HEPES-NMDG, 3.8 µM $[SrCl_2]_{free}$, pH 7.6
C, Black trace	150 mM NaCl, 1 mM EGTA-NMDG, 1 mM EDTA-NMDG, 20 mM HEPES-NMDG, pH 7.6	150 mM NaCl, 1 mM EGTA-NMDG, 1 mM EDTA-NMDG, 20 mM HEPES-NMDG, pH 7.6
C, Navy trace	150 mM NaCl, 1 mM EGTA-NMDG, 1 mM EDTA-NMDG, 20 mM HEPES-NMDG, 320 nM $[SrCl_2]_{free}$, pH 7.6	150 mM NaCl, 1 mM EGTA-NMDG, 1 mM EDTA-NMDG, 20 mM HEPES-NMDG, 320 nM $[SrCl_2]_{free}$, pH 7.6
C, Red trace	150 mM NaCl, 1 mM EGTA-NMDG, 1 mM EDTA-NMDG, 20 mM HEPES-NMDG, 6.3 µM $[SrCl_2]_{free}$, pH 7.6	150 mM NaCl, 1 mM EGTA-NMDG, 1 mM EDTA-NMDG, 20 mM HEPES-NMDG, 6.3 µM $[SrCl_2]_{free}$, pH 7.6
D-F	150 mM NaCl, 1 mM EGTA-NMDG, 1 mM EDTA-NMDG, 20 mM HEPES-NMDG, 0-6.3 µM $[SrCl_2]_{free}$, pH 7.6	150 mM NaCl, 1 mM EGTA-NMDG, 1 mM EDTA-NMDG, 20 mM HEPES-NMDG, 0-6.3 µM $[SrCl_2]_{free}$, pH 7.6

Table S6. Buffer compositions for Figure 6 (Noise analysis of Na^+ currents to deduce single channel properties). For $[Ca^{2+}]_{free}$ and $[Sr^{2+}]_{free}$, MaxChelator was used to determine the amount of $CaCl_2$ or $SrCl_2$ added to reach the desired value (Methods).

Panel	Trans Chamber	Cis Chamber
A, Black trace	40 mM $CaCl_2$, 20 mM HEPES-NMDG, pH 7.6	40 mM $CaCl_2$, 150 mM NaCl, 1 mM EGTA-NMDG, 1 mM EDTA-NMDG, 20 mM HEPES-NMDG, pH 7.6
A, Navy trace	40 mM $CaCl_2$, 50 nM $La(NO_3)_3$, 20 mM HEPES-NMDG, pH 7.6	40 mM $CaCl_2$, 150 mM NaCl, 1 mM EGTA-NMDG, 1 mM EDTA-NMDG, 20 mM HEPES-NMDG, pH 7.6
A, Red trace	40 mM $CaCl_2$, 10 µM $La(NO_3)_3$, 20 mM HEPES-NMDG, pH 7.6	40 mM $CaCl_2$, 150 mM NaCl, 1 mM EGTA-NMDG, 1 mM EDTA-NMDG, 20 mM HEPES-NMDG, pH 7.6
C	40 mM $CaCl_2$, 50 nM $La(NO_3)_3$, 20 mM HEPES-NMDG, pH 7.6	40 mM $CaCl_2$, 150 mM NaCl, 1 mM EGTA-NMDG, 1 mM EDTA-NMDG, 20 mM HEPES-NMDG, pH 7.6
D	40 mM $CaCl_2$, 0-10 µM $La(NO_3)_3$, 20 mM HEPES-NMDG, pH 7.6	40 mM $CaCl_2$, 150 mM NaCl, 1 mM EGTA-NMDG, 1 mM EDTA-NMDG, 20 mM HEPES-NMDG, pH 7.6

Table S7. Buffer compositions for Figure 7 (Noise analysis of Ca^{2+} currents).

Panel	Trans Chamber	Cis Chamber
A-C, Black curve	40 mM $CaCl_2$, 1 mM EGTA-NMDG, 1 mM EDTA-NMDG, 20 mM HEPES-NMDG, pH 7.6	40 mM $CaCl_2$, 150 mM NaCl, 1 mM EGTA-NMDG, 1 mM EDTA-NMDG, 20 mM HEPES-NMDG, pH 7.6
A-C, Cyan trace	28 mM $SrCl_2$, 12 mM $CaCl_2$, 1 mM EGTA-NMDG, 1 mM EDTA-NMDG, 20 mM HEPES-NMDG, pH 7.6	40 mM $CaCl_2$, 150 mM NaCl, 1 mM EGTA-NMDG, 1 mM EDTA-NMDG, 20 mM HEPES-NMDG, pH 7.6
A-C, Red trace	40 mM $SrCl_2$, 1 mM EGTA-NMDG, 1 mM EDTA-NMDG, 20 mM HEPES-NMDG, pH 7.6	40 mM $CaCl_2$, 150 mM NaCl, 1 mM EGTA-NMDG, 1 mM EDTA-NMDG, 20 mM HEPES-NMDG, pH 7.6
D-F, 0.0 Sr^{2+}	40 mM $CaCl_2$, 1 mM EGTA-NMDG, 1 mM EDTA-NMDG, 20 mM HEPES-NMDG, pH 7.6	40 mM $CaCl_2$, 150 mM NaCl, 1 mM EGTA-NMDG, 1 mM EDTA-NMDG, 20 mM HEPES-NMDG, pH 7.6
D-F, 0.15 Sr^{2+}	6 mM $SrCl_2$, 34 mM $CaCl_2$, 1 mM EGTA-NMDG, 1 mM EDTA-NMDG, 20 mM HEPES-NMDG, pH 7.6	40 mM $CaCl_2$, 150 mM NaCl, 1 mM EGTA-NMDG, 1 mM EDTA-NMDG, 20 mM HEPES-NMDG, pH 7.6
D-F, 0.30 Sr^{2+}	12 mM $SrCl_2$, 28 mM $CaCl_2$, 1 mM EGTA-NMDG, 1 mM EDTA-NMDG, 20 mM HEPES-NMDG, pH 7.6	40 mM $CaCl_2$, 150 mM NaCl, 1 mM EGTA-NMDG, 1 mM EDTA-NMDG, 20 mM HEPES-NMDG, pH 7.6
D-F, 0.5 Sr^{2+}	20 mM $SrCl_2$, 20 mM $CaCl_2$, 1 mM EGTA-NMDG, 1 mM EDTA-NMDG, 20 mM HEPES-NMDG, pH 7.6	40 mM $CaCl_2$, 150 mM NaCl, 1 mM EGTA-NMDG, 1 mM EDTA-NMDG, 20 mM HEPES-NMDG, pH 7.6
D-F, 0.70 Sr^{2+}	28 mM $SrCl_2$, 12 mM $CaCl_2$, 1 mM EGTA-NMDG, 1 mM EDTA-NMDG, 20 mM HEPES-NMDG, pH 7.6	40 mM $CaCl_2$, 150 mM NaCl, 1 mM EGTA-NMDG, 1 mM EDTA-NMDG, 20 mM HEPES-NMDG, pH 7.6
D-F, 0.85 Sr^{2+}	34 mM $SrCl_2$, 6 mM $CaCl_2$, 1 mM EGTA-NMDG, 1 mM EDTA-NMDG, 20 mM HEPES-NMDG, pH 7.6	40 mM $CaCl_2$, 150 mM NaCl, 1 mM EGTA-NMDG, 1 mM EDTA-NMDG, 20 mM HEPES-NMDG, pH 7.6
D-F, 1.0 Sr^{2+}	40 mM $SrCl_2$, 1 mM EGTA-NMDG, 1 mM EDTA-NMDG, 20 mM HEPES-NMDG, pH 7.6	40 mM $CaCl_2$, 150 mM NaCl, 1 mM EGTA-NMDG, 1 mM EDTA-NMDG, 20 mM HEPES-NMDG, pH 7.6

Table S8. Buffer compositions for Figure 8 (An anomalous mole fraction effect identifies ion-ion interactions in the pore).

Panel	Trans Chamber	Cis Chamber
A & B	150 mM NaCl, 1 mM EGTA-NMDG, 1 mM EDTA-NMDG, 20 mM HEPES-NMDG, 0-40 mM $CaCl_2$, pH 7.6	150 mM NaCl, 1 mM EGTA-NMDG, 1 mM EDTA-NMDG, 20 mM HEPES-NMDG, 0-40 mM $CaCl_2$, pH 7.6

Table S9. Buffer compositions for Figure 9 (Ca^{2+} saturation).
